# Colorectal Cancer: From the Genetic Model to Posttranscriptional Regulation by Noncoding RNAs

**DOI:** 10.1155/2017/7354260

**Published:** 2017-05-10

**Authors:** María Antonia Lizarbe, Jorge Calle-Espinosa, Eva Fernández-Lizarbe, Sara Fernández-Lizarbe, Miguel Ángel Robles, Nieves Olmo, Javier Turnay

**Affiliations:** ^1^Departamento de Bioquímica y Biología Molecular I, Facultad de Ciencias Químicas, Universidad Complutense, 28040 Madrid, Spain; ^2^Departamento de Oncología Radioterápica, Hospital Universitario Ramón y Cajal, 28034 Madrid, Spain

## Abstract

Colorectal cancer is the third most common form of cancer in developed countries and, despite the improvements achieved in its treatment options, remains as one of the main causes of cancer-related death. In this review, we first focus on colorectal carcinogenesis and on the genetic and epigenetic alterations involved. In addition, noncoding RNAs have been shown to be important regulators of gene expression. We present a general overview of what is known about these molecules and their role and dysregulation in cancer, with a special focus on the biogenesis, characteristics, and function of microRNAs. These molecules are important regulators of carcinogenesis, progression, invasion, angiogenesis, and metastases in cancer, including colorectal cancer. For this reason, miRNAs can be used as potential biomarkers for diagnosis, prognosis, and efficacy of chemotherapeutic treatments, or even as therapeutic agents, or as targets by themselves. Thus, this review highlights the importance of miRNAs in the development, progression, diagnosis, and therapy of colorectal cancer and summarizes current therapeutic approaches for the treatment of colorectal cancer.

## 1. Introduction

Colorectal cancer (CRC) is a major global cause of morbidity and mortality in developed countries. CRC is a heterogeneous disease regarding tumor localization and genetic and racial differences, and multiple interactions with environmental factors, diet, and style of life influence its development. CRC risk factors include hereditary components (i.e., hereditary nonpolyposis colorectal cancer), predisposal to polyp formation, large bowel inflammatory diseases, obesity, high fat diet, alcoholism, smoking, and stress [1–5]. The use of biomarkers to predict the risk for CRC or the existence of early stages of the tumor could contribute to decreasing the development of the disease and allowing early intervention. In this review, we give an overview of colorectal carcinogenesis mechanisms and therapies that are currently being used. Among the multiple factors involved in CRC development and progression, in the last years, the noncoding RNA molecules have been identified as important factors regulating many biological pathways. For this reason, we also focus on the regulatory effects of noncoding RNAs and their role in CRC development. Among these RNA molecules, microRNA (miRNAs) expression dysregulation has been reported as an important factor in the development of CRC. Moreover, they can also be considered as good biomarkers for the early detection of CRC in plasma or stool, as well as predictors of therapy efficacy and as promising tools or targets for new therapeutic treatments. In addition, other noncoding RNAs (long noncoding RNAs and circular RNAs) may play important roles either in CRC-related gene regulation or in acting as miRNA regulators.

In this review, we first briefly analyze the colonic crypt organization and the mechanisms of colorectal carcinogenesis, followed by an initial description of the different types of noncoding RNAs as new molecules involved in the regulation of gene expression. We then analyze the main type of regulatory RNAs, miRNAs. First, we introduce their general characteristics and their biogenesis pathway that leads to biologically active miRNA-induced silencing complexes (miRISC). Later on, we present the current knowledge on the involvement of noncoding RNAs in CRC, beginning with the dysregulation of the expression of specific miRNAs, the main signaling pathways involved, and the importance of miRNA polymorphisms in CRC risk. The role of long noncoding RNAs and circular RNAs in CRC is also discussed, with emphasis in the regulation of miRNA function. Finally, we present colorectal cancer treatments and biomarkers, discussing first the classical therapies and potential biomarkers to predict the response to these treatments, followed by an analysis of the use of miRNAs as diagnostic and prognostic markers, their involvement in chemo- and radioresistance, and ending by discussing their potential use as therapeutic targets and tools in CRC.

## 2. Colonic Crypt Organization

The adult colonic epithelium, a single sheet of columnar epithelial cells supported by the lamina propria, has a well-defined architecture organized into crypts, which are dynamic structures that are constantly self-renewing (it is replaced every five days) [16]. The homeostatic self-renewal of the intestine depends on a complex network of interplay involving many cellular processes, including proliferation, differentiation, migration, and apoptosis. All these phenomena are finely coordinated by different signaling pathways (Wnt, Notch, Ephrin, or antagonists of BMP), playing a critical role in the Wnt signaling cascade. Wnt signaling ligands are thought to be produced by mesenchymal cells of the myofibroblast lineage closely attached to the basal lamina that surrounds the crypt.

Three differentiated epithelial lineages mediate colonic function: the colonocytes, or absorptive enterocytes, the mucus-secreting goblet cells, and the less abundant enteroendocrine cells. Each crypt unit is maintained by multipotent stem cells (SCs), located at the bottom of the crypt. During asymmetric division, SCs undergo self-renewal and generate a population of transit-amplifying cells, or progenitors, that, upon migration upwards the crypt, proliferate and differentiate into one of the epithelial cell types of the intestinal wall. These cells, enterocytes, goblet cells, and enteroendocrine cells, continue migrating upwards along the villus until they reach the villus tip where they undergo apoptosis and are shed into the lumen of the intestine [17].

Transit-amplifying cells and stem cells occupy the lower two-thirds of colonic crypts [16]. The terminally differentiated cells, which are found in the top third of the crypt, are continually extruded into the lumen. Adult SCs are defined by self-renewal, potential for multilineage differentiation, and tissue regeneration [18]. The “stem cell zone” model states that small undifferentiated cycling cells (the crypt base columnar cells) are the true intestinal SCs [17].

## 3. Colorectal Carcinogenesis

Colorectal cancer, attending to incidence and mortality statistics, is the third most common form of cancer in men and the second among women. It is one of the main causes of cancer-related death in the more developed countries, leading to an incidence of 1.4 million cases and 693,900 CRC-related deaths occurred in 2012 [19] (http://globocan.iarc.fr). The incidence and mortality from CRC are markedly lower in the less developed countries. Epidemiological studies have shown incidence variations between areas that may be partially explained by varied cultures and lifestyles in different countries and regions. Factors as the lack of dietary fibers, smoking, overweight, obesity, physical inactivity, red and processed meat consumption, and excessive alcohol intake are potential risks for CRC. Around a quarter of CRC patients are incurable at diagnosis and half of those who undergo potentially curative surgery will ultimately develop metastatic disease. Despite the emergence of new targeted agents, early CRC screening, and the use of improved treatments and various therapeutic combinations, none of the available treatments is curative in patients with advanced cancer. Thus, although the application of some medical procedures, such as the use of colonoscopies or the fecal occult blood test, contribute greatly to the early diagnosis and CRC treatment, the deaths from CRC have decreased in several western countries, but its mortality increased in others, such as some East Asian or Latin American countries.

CRC is a multifactorial disease, a complex multistep process involving genetic background, numerous genetic alterations, and environmental influences [5]. Tumors are formed by a heterogenic pool of cells with distinct differentiation patterns. Several studies have been directed towards the identification of markers associated with the initiation and progression of this type of tumors, which normally involve multisequential steps along the adenoma-to-carcinoma transition. The accumulation of mutations in key genes, involving the inactivation of tumor suppressor genes and oncogene activation, follows the progression of the disease. Histopathological and molecular analyses are conducted by Fearon and Vogelstein to formulate, in the 1990s, the “adenoma-carcinoma model” or sequence of tumor progression [20]. Thus, the neoplastic process, initiated by* APC (Adenomatous Polyposis Coli)* or* CTNNB1 (β-catenin)* mutations, results from the sequential mutation of other genes, such as* KRAS* and* TP53*, in the context of a growing genomic instability.

The mechanisms of colorectal carcinogenesis have been extensively described [1, 3–5, 21]. The two major types of genomic instability found in colorectal cancers are chromosomal instability (CIN) and microsatellite instability (MSI). CIN is often associated with mutational inactivation of* APC* gene. The inactivation of* APC* is found in about 85% of sporadic CRC and is mutated in the germline of patients with FAP* (Familial Adenomatous Polyposis)*. This pathway often includes activation of oncogenes such as* COX2* and* KRAS* and inactivation of additional tumor suppressor genes such as* DCC/DPC4 (Deleted in Colon Cancer/Deleted in Pancreatic Cancer, locus 4)* and* TP53*.

MSI is due to defective/inactivation of DNA mismatch repair* (MMR)* genes. Mutations in these genes cause genetic defects in other genes that are involved in growth control and behave like tumor suppressor genes. Lynch syndrome and hereditary nonpolyposis colorectal cancer (HNPCC) syndrome are due to inherited mutations in one of the mismatch repair genes (including* MLH1*,* MSH2*, and* PMS2*).* MMR* mutations are found in approximately the remaining 15% of sporadic CRC [1, 3]. In the CpG island methylator phenotype, a number of genes become transcriptionally silenced because of hypermethylation of their promoters, and this represents a key epigenetic mechanism of inactivation of tumor suppressor genes, including certain DNA repair genes [1].

The progression of carcinomas to invasive and metastatic disease may involve localized occurrences of a process known as the epithelial-mesenchymal transition (EMT). EMT results in epithelial cells becoming spindle shaped, with loss of cellular polarity similar to mesenchymal cells. These phenotypic changes correlate with increased cellular motility and invasion ability, more characteristic of mesenchymal cells. EMT in metastatic colorectal carcinoma has been studied to identify molecular events that contribute to disease progression [22]. Although loss of E-cadherin function is an initial event in EMT, the expression of specific integrins such as *α*5*β*6 as a consequence of EMT enables invasive cells to interact with interstitial matrices and to sustain activation of TGF-*β* (Transforming Growth Factor-*β*). EMT also induces autocrine signaling involving VEGF and Flt-1 (its receptor) enabling invasive cells to become self-sufficient for survival. Recent research has demonstrated a convincing link between EMT and cancer stem cells (CSC) as well as their association with CRC progression and therapeutic resistance [23].

It is well known that tumors are composed by a heterogeneous population of cells differing in morphology, marker expression, proliferation ability, and tumorigenic potential. But, what is the origin of the tumor cells? Traditional models of carcinogenesis assumed that this heterogeneity could be explained by stochastic genetic events and microenvironmental influence leading to clonal selection. However, the stochastic theory for the cellular origin of cancer, based upon the assumption that all cancer cells are equally malignant and able to give rise to tumors, has been changed in favor of the hierarchical theory. The latter assumes that tumor cells are hierarchically organized and only a rare subpopulation of undifferentiated cells at the apex of this hierarchy have the unique biological properties necessary for tumor initiation, maintenance, and spreading [24]. Evidence is increasingly supporting the idea that human cancers can be considered as a stem cell disease. According to the CSC model, malignancies originate from a small fraction of cancer cells that show self-renewal and pluripotency and are capable of initiating and sustaining tumor growth [25]. In this model, the stem cell-like cells (CSC or tumor-initiating cells) are capable of propagating a tumor in the same way as normal stem cells control proliferation and differentiation in normal tissue [18]. In fact, it is now accepted that CSC can originate from mutations in normal somatic stem cells that deregulate their physiological programs. Alternatively, mutations may target more committed progenitor cells or even mature cells, which become reprogrammed to acquire stem-like functions. In any case, mutated genes should promote expansion of stem/progenitor cells, thus increasing their predisposition to cancer development by expanding self-renewal and pluripotency over their normal tendency towards relative quiescence and proper differentiation [26].

In summary, recent evidence points out that cancer can be considered a disease in which mutations either convert normal stem cells into aberrant counterparts or induce more differentiated cells to revert towards a stem cell-like behavior. This has major implications for the development of new targeted therapeutic strategies aimed at eradicating the tumor stem cell population. In fact, CSCs exhibit higher resistance to cytotoxic drugs and radiation as compared to bulk tumor cells. Classical therapeutic approaches may preferentially kill more differentiated cells while sparing CSCs. Survival of even few CSCs would later result in subsequent tumor regrowth and disease relapse [27].

The identification CSC populations in human colon tumors was first published in 2007 by two independent groups [28, 29]. This subset of cells, identified as CD133^+^, was able to initiate tumor growth in murine xenograft models (~1 in 262 CD133^+^ colon cancer cells represented a CSC) [28]. The expression of CD133 has also been demonstrated in some tumor cell lines, including Caco-2 cells from human colon adenocarcinoma [30], and in colorectal tumors and cells derived from them [28, 29]. Moreover, CD44 and CD166 expression can also be used to enrich for a CSC subset in colon cancers [31]. The use of several cell-surface markers, such as CD44, CD24, CD133, CD166, EpCAM (epithelial cell adhesion molecule), and ABCB5 (ATP-binding cassette subfamily B member 5), has successfully allowed the isolation of CSCs from solid tumors, including human colon, breast, brain, pancreatic, liver, ovarian, and melanoma. A number of markers used in cell sorting are emerging as being predictive of disease progression, indicating that they identify clinically important cell populations.

Other aspect that was considered in CRC is gene hypomethylation, which was first described in 1983 by Feinberg and Vogelstein [32]. Since then, several epigenetic abnormalities have been reported, mainly in the first stages of CRC development, while only few have been found to happen at more advanced stages (adenocarcinoma and metastasis) [33, 34]. Hypermethylation at gene promoter or transcription start region has been described to be cause of gene transcription silencing during CRC development, and many of the affected genes normally exhibited tumor suppressor roles. In contrast, hypomethylation activates genes that are normally silenced and results in global chromosomal instability [35]. Altered acetylation or methylation of histones has also been reported in CRC affecting the expression of tumor-associated genes and cell cycle progression. However, this epigenetic modification is gene-specific and a general pattern has not been found. HDAC inhibitors as butyrate, TSA (Trichostatin A), or SAHA (suberanilohydroxamic acid, also known as Vorinostat) induce not only hyperacetylation but also hypermethylation of histones altering the expression of genes; among them, several ones are involved in tumor development and cell cycle and arresting cells in G_1_ phase [36–39]. Some cells may even become resistant to HDAC inhibitors and develop an overall resistance to different types of stress enhancing their malignant potential, as has been described for colon carcinoma cells [40–44]. Additionally, SC may become CSC under a series of epigenetic and genetic alterations. Both hypermethylation and hypomethylation of key genes are involved in epigenetic changes during SC malignant transformation [35]. Moreover, the fully differentiated somatic cells can also acquire CSC-characteristics through massive genetic and epigenetic reprogramming. The CSCs then give rise to and maintain the heterogeneous tumor mass [45].

## 4. New Players in Gene Expression Regulation: Noncoding RNAs

### 4.1. The Discovery of the Noncoding RNAs

In humans, about 2-3% of transcripts have the capacity to encode protein and approximately 20,000–25,0000 genes encode proteins, whereas many noncoding elements are transcribed into noncoding RNA (ncRNA). However, functional ncRNAs may arise from only a small fraction of the total genome. These ncRNAs are a class of RNA molecules that are involved in the regulation of biological processes, including gene expression, epigenetic processes, cell differentiation, proliferation, migration and apoptosis, and transcriptional and posttranscriptional regulation, and are implicated in different human diseases. Due to the role of ncRNAs in such key processes, they are a focus of research interest and several excellent reviews on this topic have been published [9, 46–49]. However, further research efforts are necessary to explore the regulatory functions of this novel class of RNA molecules.

In the 1970s, researchers began to realize that the genome was transcribed into nonprotein-coding RNAs, as the known housekeeping ribosomal and transfer RNAs (rRNAs and tRNAs), and the role of other ncRNAs in gene expression regulation was postulated. In the 1980s, small nucleolar RNAs (snoRNAs; intermediate-sized ncRNAs of 60–300 nt) and small nuclear RNAs (snRNAs; average length approximately 150 nt) became recognized as major players in posttranscriptional RNA processing. When were ncRNAs first described? Introns, that were discovered in 1977 [50], accounted for a part of the noncoding sequences. However, for more than a decade, researchers paid very little attention to what happened with the intronic RNA fragments after their removal from the pre-mRNA. It was not until 1990 that Liu and Maxwell described that the intronic sequences of the mouse hsc70 heat shock gene* (HSPA8)* were the source for U14 snoRNA [51]. These molecules are primarily involved in chemical modifications of other RNAs, mainly rRNAs, tRNAs, and snRNAs. In this process, the complementary pairing of their guide sequences with that of the RNA target is essential. There are two main classes of snoRNA, the C/D box snoRNAs, which are associated with methylation, and the H/ACA box snoRNAs, which are associated with pseudouridylation. snoRNAs are components of the small nucleolar ribonucleoproteins (snoRNPs), which are complexes responsible for the two aforementioned sequence-specific modifications: 2′-*O*-methylation and pseudouridylation. Many of the newly discovered snoRNAs are synthesized by an intron-processing pathway, which provides a potential mechanism for coordinating nuclear RNA synthesis. For example, posttranscriptional modifications of rRNAs take place in the nucleolus (the nuclear compartment within which ribosomes are formed) and facilitate rRNA folding and stability [52].

The functions of ncRNAs are quite variable and specific. For example, studies on snRNAs have shown that their primary function is the processing of pre-mRNAs (heterogeneous nuclear RNA or hnRNA) in the nucleus. The 7SK-RNA plays a role in transcription regulation by controlling the positive transcription elongation factor P-TEFb. It is found in a small nuclear ribonucleoprotein complex (snRNP) with a number of other proteins that regulate the stability and function of the complex. On the other hand, the 7SL-RNA is a component of the eukaryotic signal recognition particle together with six distinct polypeptides. The ribozyme ribonuclease P, that cuts the leader 5′sequences of all tRNAs, is an example of catalytic RNA. The impact of all of these topics in which ncRNAs are involved has been reflected in three Nobel Prizes. In 1989, the Nobel Prize in Chemistry was awarded jointly to Sidney Altman and Thomas R. Cech* “for their discovery of catalytic properties of RNA*.*”* In 2006, the Nobel Prize in Physiology or Medicine was awarded jointly to Andrew Z. Fire and Craig C. Mello* “for their discovery of RNA interference - gene silencing by double-stranded RNA*.*”* In 2009, the Nobel Prize in Chemistry was awarded jointly to Venkatraman Ramakrishnan, Thomas A. Steitz, and Ada E. Yonath* “for studies of the structure and function of the ribosome*.*”*

According to size, the ncRNAs have been systematically classified into two groups, small ncRNAs (sncRNAs) and long ncRNAs (lncRNAs), which are shorter than 200 or longer than 200 nt, respectively. The sncRNAs can be divided into microRNAs (miRNAs), small interfering RNAs (siRNAs), snoRNAs, snRNAs, PIWI-interacting RNAs (piRNAs), ribozymes, and telomere-associated RNAs (TERC), circular RNAs (circRNAs), and other sncRNAs. The classification of lncRNAs is based on different parameters, as genomic location, effect exerted on DNA sequences, mechanism of action, and their targeting mechanism [47]. In addition, they can be classified according to their position in the genome into intergenic (lincRNA), intronic, bidirectional, sense, and antisense lncRNAs. Several studies have revealed that both small and long ncRNAs can regulate gene expression at the transcriptional, posttranscriptional, and epigenetic levels. Among the former, miRNAs, endogenous siRNAs, and piRNAs have been described to exert a gene regulation role. Although only a few lncRNAs have been characterized to date, recent work has revealed the regulatory role of lncRNAs. In addition to their length, known lncRNAs have key differences with small ncRNAs and some intriguing similarities to mRNAs. As we describe later on, small ncRNAs regulate gene expression by sequence-specific binding, but lncRNAs do it through diverse mechanisms that remain unclear [9, 53].

In 1998, the Nobel Laureates Andrew Fire and Craig Mello described a key mechanism for controlling the flow of genetic information. They demonstrated the potent and specific genetic interference by exogenous double-stranded RNA in* Caenorhabditis elegans*, which was more effective in producing interference than either strand individually [54]. Such effects have been proposed to result from a simple antisense mechanism that depends on hybridization between the injected RNA and endogenous messenger RNA transcripts. In this process, gene expression is inhibited by degradation of specific mRNA molecules. The mechanism underlying RNA interference and components of the RNA silencing machinery were identified during the following years. Thus, two types of sncRNAs, siRNA and miRNA, have attracted considerable attention because they play an important role in gene regulation and have a therapeutic potential in the treatment of many different diseases including cancer [55].

In the last years, growing experimental evidences suggest that alterations in gene-regulator ncRNAs are relevant in tumorigenesis and that most human tumors are characterized by dysregulation of miRNAs. In this review, we focus on miRNAs, which are considered the main regulatory molecules among ncRNAs and have been shown to play an important role in CRC and also in the participation of lncRNAs as well as circRNAs, both regulating miRNA activity.

### 4.2. MicroRNAs: The Main Noncoding Regulatory RNAs

miRNAs are naturally occurring small noncoding, single-stranded RNA molecules of approximately 22 nucleotides in length that are derived from hairpin precursors. MiRNA are well characterized as a large class of gene regulators and are estimated to regulate the translation of more than 60% of protein-coding genes [9]. They negatively regulate gene expression by binding to complementary sequences within target mRNAs for posttranscriptional gene silencing. This binding occurs mainly within the 3′-untranslated region (3′UTR) of target mRNA based on sequence complementarity and results in target mRNA translational repression or mRNA degradation [56, 57]. In addition, some miRNAs have been shown to bind to the open reading frame or to the 5′UTR of the target mRNAs. miRNAs are a class of highly abundant noncoding RNA molecules that are involved in several biological processes, as cell proliferation and differentiation, and in many diseases including cancer.

Since the initial discovery of lin-4 and let-7 miRNAs, which are components of the gene regulatory network that controls the timing of larval development in* C. elegans*, a plethora of miRNAs has been reported. The association of miRNAs with cancer development was described for the first time after the detection of frequent deletions of miR-15 and miR-16 loci in B-cell chronic lymphocytic leukemia [7, 58]. The hallmarks in miRNA discovery and its relationship with cancer are shown in Table 1. During the last twelve years, several studies describing the correlations between miRNA and cancer and metastasis, their role as molecular biomarkers, and their involvement in the oncogene and tumor suppressor networks have been published [59].

**Figure 1:**
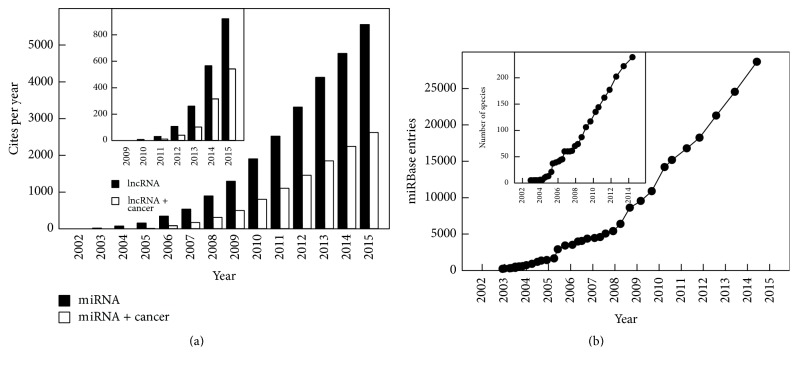
Evolution of the number of publications on miRNAs and lncRNAs. (a) The number of publications per year (searched in PubMed) that contain the terms “microRNA” (black bars) and “microRNA + cancer” (empty bars) in their title, abstract, or keywords, is shown. The inset is equivalent with the terms “lncRNA” and “lncRNA + cancer”. (b) Evolution of the number of entries in miRBase from December 2002 until the last release (release 21) I June 2014. The inset shows the increase in the number of species where miRNAs have been found.

Sequences and annotations of published miRNAs are archived in miRBase database. The first release of miRBase in 2002 contained 218 miRNA loci from five species (Table 1). Since then, the miRNA discovery field has exploded with hundreds of miRNAs found to be present in each studied animal and plant genome. They are extended to the majority of vertebrates and are conserved throughout evolution, from worms to plants and humans. The number of published miRNA sequences in miRBase continues to increase rapidly, mainly driven by small RNA deep sequencing experiments. Thousands of miRNAs across different species have been identified; the exponential evolution of the number of publications along the years is shown in Figure 1(a) as well as the increase in miRNA entries in the miRBase database and number of species where they have been found (Figure 1(b)). The latest version of the miRNA database (release 21; June 2014) contains 28,645 miRNA loci from 223 species and 16 viruses, corresponding at about 35,828 mature miRNAs. This number is even expanding and mirBase 21 contains at the moment 1,881 precursors and 2,588 human mature miRNAs representing 1–3% of all human genes [60].

**Figure 2:**
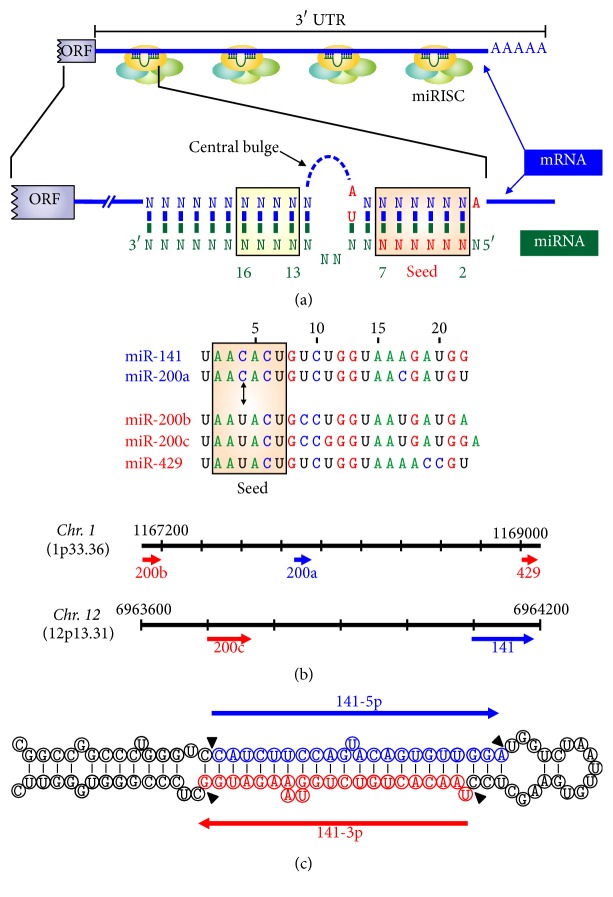
Interaction between miRNAs and target mRNAs in animals and miRNA-200 family. (a) miRNAs (green) target mRNAs (blue) in the 3′UTR. This mRNA region can bind one or various miRISC complexes arising from the same or different miRNAs. A perfect complementarity is found in the seed region between nucleotides 2 and 7 from the 5′ end of the miRNA (orange box). A central bulge prevents endonucleolytic cleavage mediated by Ago2 (a major difference with the miRNA-target mRNA interaction in plants). A few nucleotide matches in the miRNA 3′ end (especially between nucleotides 13 and 16; yellow box) are necessary for the best stabilization of the miRNA/mRNA duplex. The presence in the mRNA sequence of an A residue in position 1 and/or an A or U residue in position 9 can increase miRNA efficiency [6]. (b) The miR-200 family of miRNAs consists of two closely related subfamilies that differ in one nucleotide within the seed sequence (boxed). The five miR-200 family members are located on two different genetic loci in chromosomes 1 and 12. (c) Schematic representation of the secondary structure of the pre-miR-141 hairpin. The sequence of the mature miR-141-3p is indicated in red and the miR-141-5p (previously called miR-141^*∗*^) is in blue. Black triangles show Dicer cleavage sites due to its RNase III activity.

The miRBase also provides rules for the standard nomenclature system of the miRNAs and the criteria for identification and naming miRNAs (http://www.mirbase.org/help/nomenclature.shtml) [61, 62]. Thus, the numbering of newly identified miRNA genes is sequential and each name is preceded by abbreviated three-letter prefixes to designate the species; for humans, those letters are “hsa”* (Homo sapiens)*. The mature sequences are designated as “miR,” whereas both the gene locus and precursor hairpins (pre-miRNA) of a miRNA are identified as “mir.” However, names referring to genomic loci should be written in italics for easier differentiation from mature sequences. miRNAs with nearly identical sequences except for one or two nucleotides are annotated with an additional lowercase letters. For example, hsa-miR-200b is closely related to hsa-miR-200c (Figures 2(a) and 2(b)). Additionally, miRNAs from genes located in different genome regions that lead to an identical mature miRNA are indicated with an additional number (e.g., miR-135a-1 and miR-135a-2). When both strands of the hairpin structure of a pre-miRNA are processed as mature miRNA, an indication must be given to specify which arm generates one or the other of the two miRNAs. Such mature sequences are named followed by -5p and -3p (e.g., miR-141-5p and miR-141-3p; Figure 2(c)). Finally, when the relative abundancy clearly indicates which is the predominantly expressed miRNA, the mature sequences are assigned names of the form miR-56 (the predominant product) and miR-56^*∗*^ (from the opposite arm of the precursor and nonfunctional). However, when the data are not sufficient to determine which sequence is the predominant one, or the functionality of a miRNA is described, names like miR-141-5p (from the 5′ arm) and miR-141-3p (from the 3′ arm) are recommended. Finally, attending to the function of miRNAs, some researchers refer to specific miRNAs as ts-miRNAs (tumor suppressor), oncomiRs (oncogenic), and epi-miRNAs (subgroup that modulate the epigenetic machinery [9]).

Several studies have indicated that miRNAs could be grouped in “seed families” based on sequence homology at the 5′ end of the mature miRNA. This 5′ end is crucial for the stability and proper loading of the miRNA into the miRNA-associated multiprotein RNA-induced silencing complex (miRISC). A schematic model of one of the canonical interactions between miRNAs and 3′UTR target mRNAs is shown in Figure 2(a). A perfect complementarity is found primarily in the seed region between nucleotides 2 and 7 from the 5′ end of the miRNA. A few nucleotide matches in the miRNA 3′ end (nucleotides 13 to 16) are necessary for the best stabilization of the miRNA/mRNA target duplex [6]. Other categories of target sites, as marginal or atypical sites, have also been reported according to their structural features. Therefore, the targets of a miRNA family are likely to overlap among members. In addition, a single mRNA can be targeted by multiple miRNAs, and an individual miRNA can have more than one mRNA target. Those mRNAs that share the same miRNA response elements, or bind members from the same miRNA family, are reported to influence the expression of each other by competing for miRNA binding. Interestingly, the mRNA-miRNA interaction network is complex; single miRNA might regulate and bind to as many as two hundred mRNA targets. The proteins corresponding to these targets can be diverse in their function; they include transcription factors, secreted factors, receptors, and transporters [63]. The chromosomal localization and seed sequences corresponding to the miR-200 family members, which comprise five miRNAs, miR-200a, -200b, -200c, miR-141, and miR-429, are shown in Figure 2(b). The secondary structure of the pre-miR-141 hairpin and the origin and sequences of miR-141-5p and miR-141-3p are presented in Figure 2(c).

### 4.3. MicroRNA Biogenesis

Eukaryotic nuclear DNA encodes miRNAs and their genomic organization is diverse. They can be located within exons or introns, in intergenic sequences or produced from lariat introns [64, 65]. Exonic miRNAs are located within exons and are independently transcribed from their own promoters into precursor pri-miRNAs. When some miRNA genes overlap protein-coding genes in the antisense direction, they also have their own promoters. In mammalian cells, more than 50% of miRNAs are found within an intron of a protein-coding gene. They may be under the control of their own independent promoter or may be cotranscribed with the host gene and processed into pre-miRNAs subsequent to intron splicing. In addition, some miRNA genes are intergenic and their transcription is driven by their own upstream promoter and may be either RNA polymerase II-dependent or III-dependent. Moreover, mirtrons are small lariat intron-derived precursor miRNAs excised by the splicing machinery. Following splicing, mirtrons undergo debranching by a lariat-debranching enzyme and then fold into hairpin structures resembling precursor miRNAs. Mirtrons are transported to the cytoplasm by exportin-5/Ran-GTP following the typical miRNA-processing pathway presented below. Additionally, an individual pri-miRNA can either produce a single miRNA or contain clusters of two or more miRNAs that are processed from a long primary transcriptional unit. In this way, the largest gene cluster of human miRNAs is located in chromosome 19q13.41. This miRNA cluster (C19MC) encodesmore than 59 mature microRNAs and is exclusively expressed in the placenta and in undifferentiated cells [66].

**Figure 3:**
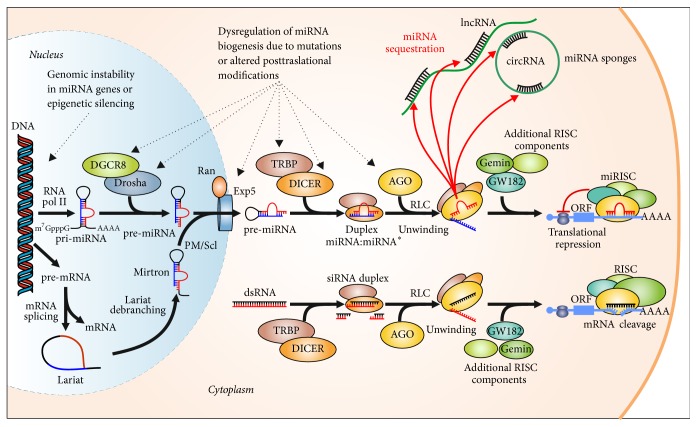
Biogenesis of miRNAs and translational repression exerted by miRNAs and siRNAs. The nascent pri-miRNA transcripts are first processed into ~70-nucleotide pre-miRNAs by Drosha/DGR8 complexes inside the nucleus. MiRNAs can also be byproducts of mRNA splicing after lariat debranching and 3′-trimming by the exosome complex PM/Scl (mirtrons). Pre-miRNAs (or mitrons) are transported to the cytoplasm by exportin 5 coupled with Ran-GTP and are processed into miRNA:miRNA^*∗*^ duplexes by Dicer/TRBP. Dicer also processes endogenous or exogenous dsRNA duplexes. Only one strand of the miRNA:miRNA^*∗*^ duplex or the siRNA duplex is preferentially assembled into RISC by the RISC loading complex (RLC). Subsequently, the RISC complex acts on its mRNA target by translational repression or mRNA cleavage, depending, at least in part, on the level of complementarity between the small RNA and its target. Alterations of miRNA function in cancer are multifactorial. They can arise from epigenetic silencing of miRNA genes or may be due to genetic instability as human microRNA genes are frequently located at fragile sites and genomic regions involved in cancers [7, 8]. Dysregulation of miRNA biogenesis machinery is also frequent in cancer mainly due to mutations in one or several of the proteins involved in processing (Drosha, DGCR8, DICER, TRBP, and Argonaute), or in nuclear export (exportin 5), or by alterations in their posttranslational modifications (PTMs) [7, 9–14]. Although specific miRNAs have been described as acting as oncogenes and tumor suppressors, the miRNA expression profile of human tumors is characterized by a general defect in miRNA production that results in global miRNA downregulation. In addition, miRNA sequestration by the so-called miRNA sponges (i.e., circRNAs and lncRNAs) can also contribute to dysregulation of miRNA function. ORF, open reading frame.

Biogenesis of miRNAs takes place through a multistep process that involves the activity of RNase III enzymes Drosha and Dicer and ultimately results in the production of mature miRNAs of about 22 nt. Several review articles summarize the miRNA biogenesis and its regulation [10–12, 65, 67–69]. The miRNA biogenesis pathway is shown in Figure 3. The process generally starts with the transcription by RNA polymerase II of the miRNA gene yielding in the nucleus primary large miRNA (pri-miRNA) transcripts which are 5′capped (m7GpppG) and 3′polyadenylated [poly(A) tail]. These long pri-miRNA transcripts are subjected to processing by the microprocessor double-stranded RNA (dsRNA) RNase III enzyme Drosha and its cofactor the dsRNA-binding protein DGCR8 (DiGeorge syndrome critical region 8). Drosha recognizes the base of the stem-loop hairpin structure and cleaves the 5′ and 3′ arms of the pri-miRNA, whereas DGCR8 directly interacts with and stabilizes the pri-miRNA and determines the precise cleavage site. Following cleavage of the pri-miRNA molecule by the Drosha microprocessor, its size is reduced to a 60–70 nt pre-miRNA precursor product (pre-miRNA) which contains an imperfect stem-loop hairpin flanked by single-stranded RNA (ssRNA). After nuclear processing, the pre-miRNAs are then exported from the nucleus into cytoplasm by exportin-5/Ran-GTP, a nuclear transport receptor complex. Dicer, another RNase III enzyme, and the transactivation-responsive RNA-binding protein (TRBP or TARBP2) process the pre-miRNA in the cytoplasm to generate a transient ~22 nt miRNA:miRNA^*∗*^ duplex with 2 nt overhangs at the 3′ ends. Next, the RISC loading complex is formed after recruiting Argonaute (AGO) protein. One miRNA strand is the antisense or guide/mature strand, while miRNA^*∗*^ is the sense or passenger strand. This duplex is then loaded into the miRISC, which includes an AGO2 protein, GW182/TNRC6 proteins, Gemin 3/4 (Dead-box helicases DDX20 and DDX42), among other potential proteins, and the mature functional guide single-stranded miRNA yielding a functional miRISC. Usually the passenger strand is cleaved. The mature functional miRNA guides the RNA-induced silencing complex (RISC) to miRNA response elements on target mRNA transcripts to posttranscriptionally negatively regulate gene expression via translation inhibition or transcript degradation [57].

An exception to this general pathway is the maturation of mirtrons. Figure 3 shows that mRNA splicing can produce miRNA-containing introns that are processed by a Drosha-independent mechanism. The lariat intron is debranched by a lariat-debranching enzyme instead of Drosha and, after refolding into hairpin structures, is transported to the cytoplasm by exportin-5/Ran-GTP and follows the typical miRNA-processing pathway. Thus, they are cleaved by Dicer and the mature strands are loaded onto miRISC. Figure 3 also shows the incorporation of the siRNAs into the RNA silencing machinery at the level of Dicer. The complex regulation of miRNA gene transcription as well as the multiple regulators (activators and repressors) of the miRNAs processing have been considered in several reviews [10, 12, 68].

**Figure 4:**
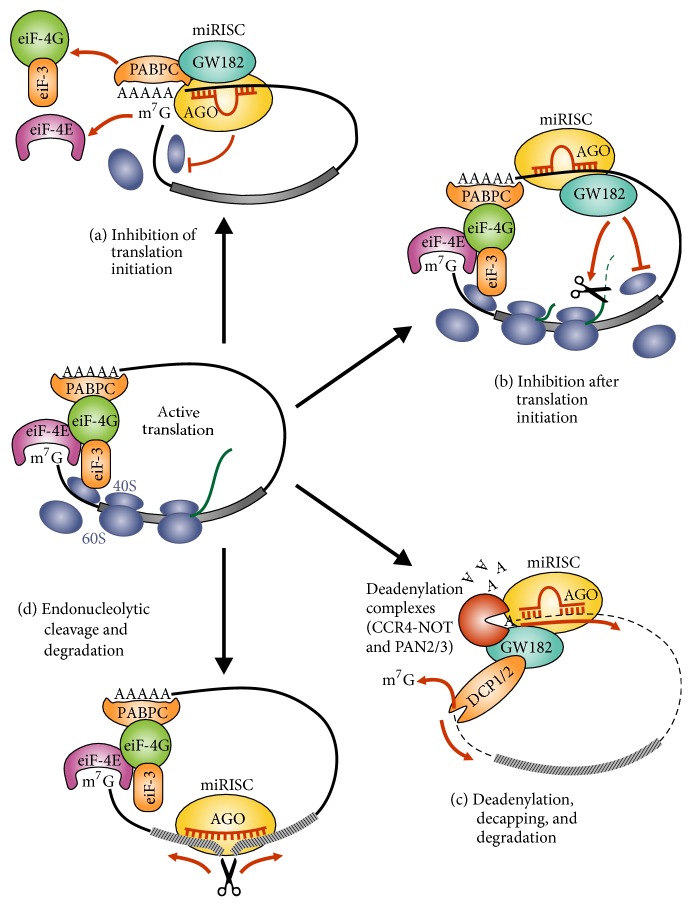
Mechanisms of target regulation by miRNAs. miRNAs regulate gene expression through multiple pathways. Eukaryotic initiation factors bind the 5′cap and the cytoplasmic poly(A)-binding protein (PABPC), connecting the 5′ and 3′ ends of mRNAs and stimulating their translation by the ribosome. (a) The miRNA-induced silencing complex (miRISC) can induce translational repression by blocking initiation; GW182 competes with eIF-4G in association with PABPC; and Argonaute (AGO) binds to the mRNA cap releasing eIF-4E, thus preventing the circularization required for efficient translation and the binding of ribosome 40S subunit to the mRNA. (b) Translational repression can also be induced by the miRISC by inhibiting a step after initiation, such as promoting ribosome drop-off or stimulating proteolysis of the nascent peptide. (c) Partial pairing of the miRNA complex to target 3′UTR sites can result in deadenylation of the mRNA by the CCR4–NOT or the PAN2/3 complexes and decapping by DCP1/2 (all of them recruited by GW182). Loss of the poly(A) tail causes dissociation of PABPC and leads to degradation of the mRNA. (d) Finally, perfect pairing between a miRNA and its target site induces endonucleolytic cleavage by AGO, leading to rapid degradation of the mRNA (occurs mainly in plants) [15].

miRNAs regulate gene expression through multiple pathways in a sequence-specific fashion. As we have already described, the degree of complementarity between the 5′seed region of the miRNA and its 3′UTR target mRNA determines the process (Figure 2(a)). In plants, the perfect or near-perfect base pairing often leads to the cleavage of target mRNAs and subsequent gene silencing by RNA interference pathway. In eukaryotic cells, also a perfect pairing between a miRNA and its target site induces endonucleolytic cleavage by Argonaute, leading to rapid degradation of the mRNA. However, the binding of miRNAs to mRNA targets with imperfect complementarity block target gene expression at the level of protein translation [12, 15, 65, 68]. In eukaryotic cells, mRNA translation is stimulated by the formation of circular structures where the 5′ and 3′ ends of mRNAs are connected through the interaction between a complex formed by the eukaryotic initiation factor eIF-4E, that binds the 5′cap, and the cytoplasmic poly(A)-binding protein (PABPC), brought together by eiF-4G. The latter also interacts with eiF-3, which binds the 40S ribosomal subunit and promotes its assembly on the mRNA (Figure 4). The partial pairing of the miRNA complex to target 3′UTR sites can result in deadenylation of the mRNA through recruitment of the CCR4–NOT or PAN2/3 complexes by the miRISC-associated GW182 proteins. Loss of the poly(A) tail causes dissociation of PABPC and leads to mRNA degradation. Moreover, the miRISC can also induce translational repression by blocking initiation via recruitment of CCR4–NOT by GW182. The translational repression can also be induced by the miRISC by inhibiting a step after initiation, such as promoting ribosome drop-off or stimulating proteolysis of the nascent peptide.

In cancer, changes in specific oncogenic and tumor suppressor miRNAs, as well as alterations in the miRNA expression profile in human tumors, have been shown to play a key role in the development of cancer. For example, let-7 family of miRNAs is downregulated in several types of cancer and is associated with poor patient outcomes [70]. However, several studies have established that mature miRNA accumulation also occurs after transcription being involved mechanisms of posttranscriptional regulation [67]. Alterations in the miRNA biogenesis pathway can also have an important role in cancer progression. In fact, it has been reported that mutations in the miRNA-processing machinery and dysregulation of miRNA biogenesis pathway are implicated in the pathogenesis of human disorders, including cancer [11]. Moreover, the core biogenesis machinery components, including Drosha, DGCR8, DICER1, and TRBP, are subject to posttranslational control such as phosphorylation and/or acetylation (reviewed in [10]). All of these dysfunctions in components of the miRNA biogenesis pathway produce expression changes in a large number of cellular miRNAs which can be correlated with poor patient outcomes [71–73].

Several genetic defects in components of the miRNA-processing machinery have been reported in cancer. Mutations in genes that encode* DROSHA, DGRC8, TARBP2, DICER1*, and* XPO5 (exportin 5)* point out the relevance of the miRNA biogenesis pathways in cellular transformation [9, 11, 13, 14, 74, 75]. For example, the export of pre-miRNAs into the cytoplasm is mediated by the exportin 5/Ran-GTP complex.* XPO5*-inactivating mutations were identified in sporadic colon, gastric, and endometrial tumors with microsatellite instability. These* XPO5* mutations impair pre-miRNA export to the cytoplasm and result in an accumulation of pre-miRNAs in the nucleus, leading to defects in miRNA biogenesis [75].

Alterations in the expression of components of the miRNA machinery are also involved in CRC. Kim and coworkers [76] analyzed the mRNA expressions of DGCR8 and AGO2 in 60 CRC tissues and adjacent histologically nonneoplastic tissues by using quantitative real-time PCR. They found that whereas mRNA expression level of DGCR8 is upregulated in CRC, the expression of AGO2 mRNA was not significantly altered in CRC tissues. Other studies using 237 samples from colorectal adenocarcinomas with moderate differentiation showed that a direct correlation between Dicer upregulation and poor prognosis in patients with CRC as also occurs on prostate cancer. On the contrary, in breast, lung, and ovary cancer, Dicer has been shown to be a marker of good prognosis [77]. It has also been reported that an increased expression of Dicer mRNA in normal mucosa from CRC patients is significantly related to poor survival independently of gender, age, tumor site, stage, and differentiation [78]. Finally, a study on the expression of Drosha, Dicer, and Ago2 mRNAs and protein in three colon cancer cell lines and in human CRC samples revealed that they were present in all the samples analyzed and the authors suggested that they were possibly implicated in CRC pathobiology. mRNA levels of Dicer were significantly augmented in stage III compared to stage II tumors suggesting that Dicer might have a role in the progression of these tumors to advanced stages [79].

## 5. Noncoding RNAs and Their Involvement in Colorectal Cancer

### 5.1. MicroRNAs and Colorectal Cancer

miRNAs are critical regulators of gene expression and an altered expression of miRNAs has been shown to be associated with various types of cancer. Figure 1(a) shows the increasing number of publications dealing with the relationship between miRNAs and cancer. More than 50% of human miRNA genes are often located in specific chromosomal regions prone to damage through deletion, amplification, or translocation, which may result in malignant transformation [7]. Cancer development can also arise from dysregulation of miRNA biogenesis pathway [74]. In the context of cancer, miRNAs may have oncogenic or tumor-suppressive roles according to the effect they have on pathways leading to tumor development. Oncogenic miRNAs, or oncomiRs, target and downregulate endogenous tumor suppressor genes, whereas tumor suppressor miRNAs play an important role in downregulating genes associated with growth and metastasis. Thus, overexpression, genetic amplification, and gain-of-function mutations of oncogenic miRNAs as well as genetic deletion and loss-of-function mutations of tumor suppressor miRNAs are linked to human cancer. Furthermore, global miRNA depletion caused by genetic and epigenetic alterations, dysregulation of components of the miRNA biogenesis machinery, or changes in global miRNA levels resulting from a defective miRNA biogenesis pathway play critical roles in the pathogenesis of human disorders, including cancer [11, 74, 220].

In CRC, some miRNAs are abnormally downregulated or upregulated; thus they may act as tumor suppressor or as oncogenes in tumor development. In addition, miRNA expression patterns have been suggested as predictive prognosis markers in CRC and support diagnosis of poorly differentiated tumors. The involvement of oncogenic and tumor suppressor miRNAs in key signaling pathways in CRC, their inductors, and targets, as well as the changes in the expression of some of them in the transition from normal colon mucosa-adenoma-carcinoma-advanced carcinoma, have been also described in many different review articles [47, 221–230]. An overview of some oncogenic and tumor suppressor miRNAs involved in CRC, their role in CRC, and the verified targets is shown in Table 2.

Accumulating evidence strongly indicates that aberrant miRNA expression is an important feature of CRC. The first association between miRNAs and CRC was described by Michael and coworkers in 2003 [87], using CRC tissue compared to healthy tissue (Table 1). Several miRNA sequences were identified, among them miR-21, miR-143, miR-145, and miR-200c. miR-143 and miR-145 exhibited significantly reduced levels of the fully processed miRNA in tumors compared to normal specimens. On the other hand, the first study that evaluated the association between miRNA expression patterns and CRC prognosis or therapeutic outcome was carried out by Schetter and coworkers [231]. miRNA microarray expression profiling of tumors and paired nontumor tissues was performed on a US test cohort of 84 patients with CRC, evaluating associations with tumor status, TNM staging, survival prognosis, and response to adjuvant chemotherapy. Associations were validated in a second, independent Chinese cohort of 113 patients using quantitative reverse transcription PCR assays. They identified 5 miRNAs which were significantly altered in CRC. In particular, miR-21 was overexpressed in 87% of colon cancers specimens and higher miR-21 expression correlated with poorer outcome.

Several studies have described the differentially expressed miRNAs in CRC. Wu and coworkers [232] measured the differential expression of miRNAs in colorectal adenocarcinoma tissues from 28 patients and analyzed their profiles at various differentiation stages. This study compared the expression level of 1547 miRNAs using qRT-PCR. Among them, 93 were found to be significantly dysregulated in colorectal adenocarcinoma relative to normal tissues. In particular, miR-1, miR-145, and miR-145^*∗*^ were downregulated more than tenfold and were suggested as potential biomarkers for CRC diagnosis. Furthermore, 58 miRNAs demonstrated significantly altered expression between well and moderately differentiated cancers, and 32 could be used to distinguish normal from cancerous tissues, as well as different levels of differentiation. In other study, microarrays were used to profile the expression of 315 human miRNAs in 10 normal mucosa samples and 49 stage II colon cancers differing with regard to microsatellite status and recurrence of disease. Several miRNAs were differentially expressed between normal tissue and tumor microsatellite subtypes, with miR-145 showing the lowest expression in cancer relative to normal tissue. Functional studies also showed that miR-145 potently suppressed growth of different colon carcinoma cell lines (LS174T, HCT116, and DLD1 cells) [233].

Hamfjord and coworkers [234] performed a global analysis of dysregulated miRNAs in paired samples of normal mucosa and tumor from eight patients with CRC. In this study, the normal and adjacent tumor tissue samples were paired, thus taking into account the baseline differences between individuals when testing for differential expression. At least, 37 miRNAs were identified as differentially expressed between the matched pairs of CRC tissues and normal colon mucosa, 19 downregulated and 18 upregulated. Some of these miRNAs were previously published as potential regulators in colorectal adenocarcinomas, such as miR-1, miR-96, and miR-145. They discovered 16 dysregulated miRNAs, which were not previously associated with colorectal carcinogenesis. The downregulated ones were miR-490-3p, miR-628-3p and miR-628-5p, miR-1297, miR-3151, miR-3163, miR-3622a-5p, and miR-3656 and the upregulated miR-105, miR-549, miR-1269, miR-1827, miR-3144-3p, miR-3177, miR-3180-3p, and miR-4326. On the other hand, a gene module-based approach to inferring key miRNAs regulating the major gene network alterations in CRC has been proposed comparing 90 normal and 107 CRC samples in total. Among the inferred candidates, three miRNAs, miR-101, miR-124, and miR-139, are frequently downregulated in CRC tumors. Computational and experimental analyses demonstrate that miR-139 can inhibit cell proliferation and cell cycle G1/S transition. In addition, miR-139 was found to be significantly downregulated in early pathological cancer stages and its expression remained at very low levels in advanced stages. MiR-139 was determined to be a key tumor suppressor in early cancer development [235]. Dysregulation in circulating blood miRNAs is reflective of those in colorectal tissues; in fact, a triple miRNA classifier consisting in miR-193a-3p, miR-23a, and miR-338-5p has been suggested as a potential blood biomarker for early detection of CRC [236].

### 5.2. Signaling Pathways in Colorectal Cancer Regulated by miRNAs

Considering the classic multistep colorectal carcinogenesis model proposed by Fearon and Vogelstein in 1990 [20], most CRCs progress through the sequential accumulation of molecular alterations associated with the adenoma-to-carcinoma progression. Dysregulation of a significant number of miRNAs in CRC and colon CSCs has been described in the literature [47, 221, 225, 237, 238] but special attention has been directed towards those miRNAs involved in signaling pathways and cellular processes. Among the signaling pathways that are modified in CRC carcinogenesis are those that result in activation of prosurvival, proproliferative, and metastasis (i.e., Wnt, EGFR, and TGF-*β*) as well as impairment of p53 function [34, 226]. Extensive studies evidence that miRNAs can regulate all the major pathways in CRC; briefly, this includes their impact on *β*-catenin/Wnt signaling (miR-135a/b, miR-139, miR-145, miR-17-92), proliferation (let-7 family, miR-18a, miR-21, miR-126, miR-143, miR-200c), apoptosis (miR-34a, miR-133b, miR-195), cell cycle control (miR- 34a, miR-192, miR-215, miR-675), p53 signaling (miR-34b/c), differentiation (miR-141, miR-200c), and migration and invasion (miR-126, miR-143, miR-196a, miR-200a/b/c, miR-373, miR-520c) [34].

Wnt activation signaling pathway is crucial for the regulation of stem cell activity to the intestinal crypt base and for the renewal of the epithelial cells. Activation of Wnt signaling induces cell survival and inhibits cell death and differentiation. The mutation of the tumor suppressor* APC* is one of the first events for the initiation of colorectal neoplasia. A miRNA-mediated mechanism has been described in CRC to control the expression of the* APC* gene and consequent activation of the Wnt signaling pathway. In adenocarcinomas, as well as premalignant colorectal adenomas, oncomiRs miR-135a and miR-135b are overexpressed and directly target the 3′UTR of the APC mRNA, suppress the APC expression, and activate Wnt signaling [127]. In addition, several Wnt-pathway genes contain binding sites in the 3′UTR of their corresponding mRNAs for members of the miR-34 family. The miR-34 family comprises three processed miRNAs that are encoded by two different genetic loci. MiR-34a is encoded by one transcript, and miR-34b and miR-34c arise from a common primary transcript. The members of the miR-34 family are direct p53 targets, which induce apoptosis, cell cycle arrest, and senescence [239]. *β*-Catenin binds to members of the TCF/LEF family of transcription factors whose activity is mainly regulated by the miR-34 family. In this way, miR-34 represses the activity of the TCF/LEF complex and establishes a relationship between the Wnt signaling pathway and p53 activity. Loss of p53 functions causes increased activity of Wnt signaling cascade and promotes the Snail-dependent EMT. Moreover, higher expression of oncogenic miR-21 in adenomas and CRC relative to normal tissues suggests that abnormal expression of this miRNA is an early event in the progression towards CRC. It has also been reported that miR-21 promotes cell migration and invasion by targeting the* PDCD4* (Programmed Cell Death Protein 4) and* PTEN* (phosphatase and tensin homolog) tumor suppressor genes [115, 116].

In early stages of cell transformation in CRC, an upregulation of miR-17-92 cluster has been detected. PTEN and E2Fs mRNAs were among the first validated miR-17-92 targets. Recently, Li and coworkers [240] have demonstrated a direct link between APC and the miR-17-92 cluster. APC represses miR-17-92 through inhibition of *β*-catenin. Mutation of* APC* leads to stabilization of *β*-catenin, which in turn binds to and activates the miR-17-92 promoter. Moreover, elevated level of *β*-catenin is significantly correlated with miR-19a (that belongs to the miR-17-92 family) overexpression in CRC.

miRNAs have been reported to regulate the stemness of colon CSCs mainly via regulation of important signaling pathways as Wnt/*β*-catenin and Notch, acting also on processes that involve CSCs altering the expression of cell cycle and EMT-related genes. Notch and Wnt pathways are involved in crypt development and in proliferation and self-renewal of colon SCs, decreasing apoptosis; these pathways are commonly upregulated in SC populations of colon cancer tissue. The most frequently reported miRNAs involved in the regulation of colon CSCs are miR-21 and miR-34a through modifications in the Wnt/*β*-catenin and Notch/C-kit-Erk pathways, respectively. miRNA-21 is overexpressed in colon CSCs and is able to downregulate the expression of* TGFBR2* gene (TGF-*β* receptor II), resulting in activation of the Wnt/*β*-catenin pathway and consequent increase in the expression of downstream target genes as* c-Myc* and* cyclin-D1* [117]. miR-21 also targets PTEN reducing its expression and with a concomitant activation of Akt signaling pathway, thus increasing the tumorigenic properties of colonic CSCs [241]. Finally, miR-21 can also control the expression of other miRNA, miR-145 and, in turn, increase the expression and activity of intact K-Ras in colon CSCs [242]. miR-34a is downregulated in colonic CSCs and restoration of its expression leads to differentiation of CSCs derived from colon cancer tissue to non-CSCs. miR-34a targets Notch signaling pathway, that plays an important role in symmetrical and asymmetrical cell division of stem cells. Moreover, c-kit (stem cell factor receptor) is a direct mRNA target of miR-34a; thus, downregulation of miR-34a leads to overexpression of c-kit and activation of several stemness markers [238].

The epidermal growth factor receptor (EGFR) signaling pathway is also involved in the development of CRC. EGFR can activate different signal transduction pathways as Ras/MAPK and the phosphatidylinositide 3 kinase- (PI3K-) Akt pathways. Activating* K-Ras* mutations have been described to induce activation of various downstream effectors that mediate tumor growth, survival, and metastasis in many cancers [34, 226]. In CRC, tumor suppressors, miR-143 and miR-145, that are located on chromosome 5, are reduced. Downregulated expression of miR-143 and miR-145 has been described to target multiple mRNAs related to the MAPK signaling pathway, including* K-Ras*,* ERK5*,* IRS-1* (insulin receptor substrate 1), all of them involved in the transition of an early adenoma to advanced stages [153, 226]. Furthermore, PTEN, a dominant negative regulator of the Akt, has been shown to be the target of different miRNAs, such as miR-21, miR-32, miR-92a, and miR-181a (Table 2). Other signaling pathways involve TGF-*β* family that regulates cellular processes such as proliferation, differentiation, apoptosis, and migration. Several miRNAs have been described to regulate TGFBR2, such as miR-17-5p, miR-20a, miR-21, miR-23b, miR-106a, and miR-301a. Particularly, miR-21, activated by the Wnt signaling pathway, is involved in stemness by regulating TGFBR2 signaling [243]. The oncogenic miR-17-92 cluster, that is modulated by c-Myc, represses TGF-*β* responses by silencing* TGFBR2* and* SMAD4*.

The tumor suppressor p53 is also involved in CRC by modulation of the response to different stress signals and controlling processes as senescence, cell cycle arrest, apoptosis, invasion, and metastasis [221, 226, 239]. Bioinformatics sequence analyses propose that up to 46% of the miRNA potential promoters contain a p53-binding site [215]. Several miRNAs are related to p53, among them are the following: let-7i, miR-20a, miR-21, miR-25, miR-34a/b/c, miR-145, miR-181b, miR- 183, miR-195, miR-215, and miR-451. Interestingly, the miR-34 family is p53-inducible; miR-34a is upregulated in CRC patients. A positive feedback loop between miR-34a and p53 has been proposed as miR-34a activates p53 by targeting sirtuin mRNA, a key regulator of p53 activity. Moreover, miR-34a downregulates transcription factor E2F and upregulates p53 in several cancers such as CRC [221].

EMT, that plays an important role in tumor progression, invasiveness, and therapeutic failure, is also regulated by Wnt and Notch signaling pathways through increasing levels of *β*-catenin that translocates to the nucleus and ultimately induces specific genes essential for EMT triggering by Wnt or by activation of NF-*κ*B or TGF-*β* pathways by Notch. Several EMT-associated miRNAs are involved in CRC such as the p53 responsive miR-200 family (miR-200a/b/c, miR-141, and miR-429), whose downregulation is believed to be an essential feature of EMT. In addition, miR-146a and miR-203 are also involved in colon CSC regulation via EMT signaling pathways. The first one is upregulated by the EMT inducer Snail that causes the opposite effect on miR-203. miR-200a and miR-200c are also downregulated in CSCs, allowing the expression of EMT inducers Zeb1 and Zeb2 with a concomitant decrease in E-cadherin levels and increased expression of stem cell markers CD166 and CD133 [162]. Other miRNAs have been described as important for the maintenance of the cancer stem cell phenotype. miR-215 and miR-140 are involved in chemoresistance to methotrexate and 5-FU treatments and in differentiation of CSCs derived from HCT-116 cells (CD133^+^, CD44^+^) possibly by targeting cell cycle and differentiation related genes [183, 185, 238]. Similarly, a population of colon CSCs expressing a CD133 surface phenotype from human HT29 colonic adenocarcinoma cells showed overexpression of 11 and downregulation of 8 miRNAs which could be potentially involved in the regulation of stem cell differentiation [244].

As mentioned earlier, the miR-34 family is p53-inducible and miR-34a is upregulated in CRC patients. However, miR-34b/c expression is not detectable in several CRC cell lines probably due to an epigenetic silencing of these miRNAs [245]. Finally, miRNAs also are regulators of the cancer epigenome; DNMTs, HDACs, and HMTs are targets of miRNAs [246]. DNMT3a is downregulated in CRC and is the target of miR-143. Similarly, miR-342 and miR-185 interact with the 3′UTR of DNMT1 mRNA [169]. Another example is miR-140; its overexpression inhibits cell proliferation of colon cancer HCT 116 (wt-p53) cells but presents lower effect in HCT 116 (null-p53) cells. This miRNA induces p53 and p21 expression and produces cell cycle arrest only in cells containing wild type of p53. Histone deacetylase 4 (HDAC4) was confirmed to be one of the important targets of miR-140. In addition, the expression of endogenous miR-140 was significantly elevated in CD133(+hi)CD44(+hi) colon cancer stem-like cells that exhibit slow proliferating rate and chemoresistance [183].

### 5.3. MicroRNA Polymorphisms and CRC Risk

Single nucleotide polymorphisms (SNPs) are common variants in the human genome and have been reported to influence disease susceptibility. SNPs in miRNAs and related loci are often located in pri-miRNA and pre-miRNA sequences, seed sequences, and the 3′UTR regions of target mRNAs [247]. Thus, such polymorphisms might influence miRNA function in three ways: by altering transcription of the primary transcript, affecting pri- and pre-miRNA processing, and modifying miRNA-mRNA interactions [248]. SNPs in miRNAs may ultimately result in the alteration of their expression and/or maturation, with possible consequences for cancer development and progression. Furthermore, approximately half of miRNA genes are located in cancer-related regions [249]. Thus, variations in these sequences may result in significant functional consequences, making them ideal candidates for cancer risk prediction.

It has been reported that around 35% of analyzed CCRs are due to genetic or hereditary factors. These factors may arise from single nucleotide polymorphisms (SNPs) or additional genetic abnormalities in both coding and noncoding genes [237]. Taking into account the important role of miRNAs in the initiation and development of CRC, the occurrence of SNPs in these molecules has been investigated as they may influence mRNA function or interrupt miRNA expression, altering its sequence, the binding site to mRNA, or even its processing potentially contributing to cancer susceptibility. Therefore, miRNA polymorphisms may be used as specific markers of predisposition for CRC diagnosis and prevention.

SNPs in specific miRNAs may alter the susceptibility of a patient to develop CRC [248, 250]. SNPs have been detected in association with an increased risk of CRC affecting either miRNAs (SNPs in miR-196a, miR-149, and pre-miR-27a or in miR-257a, affecting binding to 3′UTR of MBL2 mRNA) [251–253], or the 3′UTR of an mRNA, as in CD86, that alters binding of several miRNAs (in this case, miR-184, miR-200a, miR-212a, miR-337, and miR-582) [254], or DOK3, affecting binding of miR-370 [255]. It has also been observed that specific SNPs of miRNA-processing machinery genes (as DICER or GEMIN3) may affect CRC susceptibility [256]. In addition, SNPs in miRNAs have been associated with CRC survival and recurrence, and thus they might be useful in predicting therapy response. This is the case of two SNPs (rs4919510 in miR-608 and rs213210 in miR-219-1) that were genotyped in 1083 CRC patients to evaluate their effect on clinical outcomes. Carriers of the variant T allele in rs213210 and receiving 5-FU chemotherapy were associated with a significantly worse survival and an increased risk of relapse, whereas patients carrying the G allele of rs4919510 and undergoing adjuvant chemotherapy were at decreased risk of relapse [257].

### 5.4. Long Noncoding RNAs in Colorectal Cancer and Regulation of miRNA Function

The long noncoding RNAs (lncRNAs) are mainly RNA polymerase II transcripts usually longer than 200 nucleotides. Some lncRNAs show additional similarities to mRNA, such as a 5′cap, 3′poly(A) tail, even though they lack an open reading frame and have no potential to encode protein. The number of genes contained within the gene family of lncRNAs is 214 in humans [http://www.genenames.org/cgi-bin/genefamilies/set/788]. lncRNA may be located in the nucleus, chromatin, or cytoplasm; these different localizations point out to different biological functions. Thus, lncRNAs regulate a variety of key cellular processes such as epigenetic silencing, gene transcription and translation, cell cycle and apoptosis, and cell differentiation and proliferation [9, 53]. For example, lncRNAs as Xist (X inactive-specific transcript, related to X-chromosome silencing) and HOTAIR (HOX Transcript Antisense Intergenic RNA) interact with chromatin remodeling complexes to induce changes in chromatin packaging, leading to reduced target gene expression [258]. In fact, HOTAIR was the first identified lncRNA that plays a critical oncogenic role through epigenetic regulatory mechanisms [259]. lncRNAs can also act as coactivators of transcription factors by interacting with RNA-binding proteins, and this interaction alters the localization and activity of the proteins. Additionally, lncRNA transcripts could competitively inhibit the ability of miRNAs to interact with their mRNA targets. They act as miRNA sponges sequestering miRNAs analogous to how artificial miRNA sponges function (Figure 3) [103, 260]. However, although empirical evidence supporting the hypothesis that competitive endogenous RNAs (ceRNAs) as lncRNAs, circRNAs, or pseudogenes can act as miRNA sponges, recent studies that model transcriptome-wide binding-site abundance suggest that physiological changes in expression of most individual transcripts will not compromise miRNA activity [261].

The number of research papers and reviews dealing with lncRNAs and their involvement in cancer is exponentially increasing every year. Figure 1(a) (inset) shows the evolution of the number of papers along the last years. Several reviews recapitulate the knowledge in this area [9, 46, 47, 53, 262–264]. Here we describe only the function of a reduced number of lncRNAs and mainly those that are involved in CRC.

lncRNAs exhibit unique profiles in various human cancers, reflecting disease progression and serving as a predictor of patient outcome [265]. Their roles as drivers of tumor suppressors and oncogenic function have been described in different types of cancer [266]. Several studies have highlighted the role of lncRNAs in the development of CRC and their involvement in chemoresistance of CRC cells [264, 267]. They are involved in processes related to CRC progression through stimulating or inhibiting cell proliferation, apoptosis, differentiation, invasion, and metastasis. CRC-related lncRNAs have been described to regulate gene expression by a broad range of mechanisms [267] such as (i) by epigenetic modifications (DNA methylation, histone modification, chromosomal instability, X-chromosome inactivation, and genomic imprinting), (ii) by lncRNA-miRNA interactions, (iii) by their actions as small RNAs or miRNA precursors or pseudogenes, (iv) by lncRNA-protein interactions, and (v) through their function as structural RNAs, in scaffolding ribonuclear protein complexes. In addition, as some lncRNAs have been detected in human body fluids by PCR, such as in plasma and urine, they have also been suggested as novel potential biomarkers for CRC diagnosis and prognosis as well as in the prediction of the response to therapy [268].

Interactions between lncRNAs and RNA sequences have been described as a regulatory posttranslational mechanism. In this way, lncRNAs can function as competing endogenous RNAs, miRNA sponges, or as pseudogenes to serve as decoys for miRNAs. As a result, alterations in target genes or in the biological function of the miRNAs could be expected. This mechanism of inhibition of miRNA activity was discovered in 2007 studying the phosphate homeostasis in* Arabidopsis thaliana* (Table 1). The non-protein-coding RNA IPS1 (Induced by Phosphate Starvation 1) contains a motif with sequence complementarity to miR-399, the phosphate starvation-induced miRNA. However, the pairing is interrupted by a mismatched loop at the expected miRNA cleavage site in IPS1. Thus, IPS1 RNA is not cleaved but instead sequesters miR-399 [104]. In mammalian cells, Ebert and coworkers established the name “miRNA sponges” describing the specific competitive inhibitors from transcripts expressed from strong promoters which contained multiple tandem binding sites to several miRNA seed families [103]. In this way, as we comment later on, several CRC-related lncRNAs may also regulate gene expression in CRC not only by binding to target proteins but also by binding to miRNAs and, consequently, preventing specific miRNAs from binding to their target mRNAs.

A clear example of miRNA sponge is the lncRNA HULC (Highly Upregulated in Liver Cancer) that is located on the human chromosome 6p24.3. It is formed by two exons and a 500 bp intron and contains a 3′poly(A) tail and a conserved miR-372 target site [269]. HULC binds to its target miR-372 and acts as a sponge inhibiting the binding of miR-372 to the mRNA transcript. HULC is overexpressed not only in hepatocellular carcinoma but also in hepatic CRC metastasis whereas, in normal tissues, primary CRC, or those cancers that metastasize to lymph nodes, its expression is null [270, 271]. The involvement of HULC in CRC metastasis to the liver suggests its role as a new biomarker for the evaluation of hepatic metastasis potential of CRC.

Loc285194 (or LSAMP antisense RNA 3) is a lncRNA with more than 2 kb in length, which contains 4 exons and is present in the focal region of chr3q13.31 (osteo3q13.31), which is the most altered region in osteosarcomas. It has two binding sites for miR-211 in its exon 4 and acts as a potential tumor suppressor as deduced from knockdown experiments, which showed an increased cell proliferation. It has been described that loc285194 is downregulated in colon tumor specimens compared with the normal ones and is a direct transcriptional target of p53 through the negative regulation of miR-211 [264, 272]. Interestingly, miR-211 promotes cell growth and represses loc285194 expression, thus creating a reciprocal repression feedback loop.

In addition to binding miRNA, several studies have demonstrated that lncRNAs can regulate gene expression by acting as miRNA precursors. In this way, lncRNA H19 (located in chromosome 11q15.5 and 6295 nt in length) has different roles in CRC. It functions as miRNA sponge, as a miRNA precursor, and was also described as an epigenetic regulator [264]. Thus, it is a good potential target for CRC treatment. H19 can promote EMT progression and accelerate tumor growth by acting as competing endogenous RNA for miR-138 and miR-200a in CRC [273]. Moreover, H19 can be processed to yield the precursor of miR-675 [274] and its expression is positively correlated with the level of miR-675 that is involved in the downregulation of Rb expression [275]. The sequence of mature miR-675 aligns with a sequence of the 3′-UTR of Rb mRNA, and the level of Rb protein appears to be negatively correlated with the levels of both H19 and miR-675 in human CRC cells [276].

The lncRNA MALAT1 (Metastasis-Associated Long Adenocarcinoma Transcript 1) is located in chromosome 11q13.1 and has a length of 8708 nt. It promotes cell proliferation, migration, tumor growth, and metastasis in CRC cells. MALAT1 interacts with and changes the distribution of splicing factors, such as SR (serine/arginine-rich) proteins. Depletion of MALAT1 affects the abundance, location, and activity of SR proteins and changes the alternative splicing of a series of pre-mRNAs [277].

lncRNAs can also function as diagnostic and prognostic biomarkers for CRC. CRNDE (Colorectal Neoplasia Differentially Expressed) and HOTAIR, for example, are upregulated in neoplastic tumor tissue and in the blood of CRC patients [278, 279]. The lncRNA CRNDE is highly elevated in CRC, is transcribed from chromosome 16, and interacts with components of the polycomb repressive complex 2 (PRC2) and CoREST complexes [280]. In addition, HOTAIR is a lncRNA that plays a role as an oncogenic molecule. It is located on chromosome 12q13.13, has 2.2 kb of length and 6 exons, and has a 3′poly(A) tail. Not only does HOTAIR interact with PRC2, but also it is necessary for PRC2 occupancy and histone H3 lysine-27 trimethylation of different genes. PRC2 is a histone methyltransferase that implements epigenetic silencing during different processes including cancer development [281]. Interestingly, unlike HOTAIR, the lncRNA PCAT-1 (Prostate Cancer-Associated ncRNA Transcript 1, located in the chromosome 8q24) is repressed by PRC2. PCAT-1 is upregulated in CRC specimens compared with normal tissues. There is a positive correlation between PCAT-1 expression and distant metastasis of CRC. The overall survival rate of the patients with high expression of PCAT-1 is significantly lower than those with low expression [282].

The CCAT1 (Colon Cancer-Associated Transcript 1; located in chromosome 8q24.21, 2,628 nt in length) is highly expressed in CRC compared to normal tissue. CCAT1 upregulation is present in primary CRC tumors, including precancerous polyps, lymph nodes, blood, and distant CRC metastasis [283]. Its increased expression was also correlated with the clinical stage of the patients, lymph nodes metastasis, and survival time after surgery. CCAT1 functions as an oncogene; it is located close to the transcription factor* c-Myc* gene and upregulates it, promoting tumor cell proliferation and migration. Moreover, c-Myc could induce CCAT1 transcription by directly binding to its promoter region. CCAT1 expression is upregulated in CRC cells and promotes cell proliferation and invasion. These findings suggest that c-Myc-activated lncRNA CCAT1 expression contributes to colon cancer tumorigenesis and metastatic process and could predict the clinical outcome of CRC and be a potential target for lncRNA direct therapy.

Interestingly, lncRNAs seem to be also involved in chemoresistance of CRC cells. In vitro studies revealed the downregulation of the lncRNA snaR (small NF90-associated RNA) and BACE1-AS (BACE1 antisense RNA) in 5-FU resistant CRC cells, contributing to increase viability and inhibiting apoptosis without altering the cell cycle [284]. Conversely, the overexpression of the lncRNA UCA1 (urothelial carcinoma associated 1) enhances cell proliferation and 5-FU resistance in colorectal cancer by inhibiting miR-204-5p possibly acting as a sponge for this miRNA [285].

Finally, we will consider two lncRNA examples of molecules related to p53 and CRC. The lncRNA TP53TG1 (TP53 target 1) located in chromosome 7q21.12 is critical for the correct response of p53 to DNA damage and its expression is induced by p53 under conditions of cellular stress. Diaz-Lagares and coworkers have described that TP53TG1 undergoes cancer-specific promoter hypermethylation-associated silencing. TP53TG1 binds to the multifaceted DNA/RNA-binding protein YBX1 to prevent its nuclear localization and thus the YBX1-mediated activation of oncogenes. TP53TG1 epigenetic inactivation in HCT-116 CRC cells releases the transcriptional repression of YBX1-targeted growth-promoting genes and creates a chemoresistant tumor [286]. The long intergenic noncoding RNA-p21 (lincRNA-p21; ~3.1 kb, 2-exon transcript, located near the* p21* gene) has a p53-binding motif in its promoter. It acts to repress many genes transcriptionally regulated by p53 and is aberrantly expressed in several types of cancer, including CRC. Experimental evidence using 30 CRC tissues, their adjacent normal mucosa, and several CRC cell lines showed that lincRNA-p21 decreases which contributes to the elevation of *β*-catenin in CRC. It was also observed that the expression of lincRNA-p21 increases following radiation exposure and enforced expression of the lincRNA enhances the CRC sensitivity to radiotherapy, by promoting cell apoptosis [287]. Considering the mechanism of gene regulation, the heterogeneous nuclear ribonucleoprotein K (hnRNP-K) associates with the promoters of many genes repressed by lincRNA-p21 in a lincRNA-p21 dependent manner. Dimitrova and coworkers [288] have described that lincRNA-p21 interacts with the hnRNP-K through its 5′ end and influences gene expression as a coactivator of p21 expression promoting the expression of polycomb target genes. After loss of lincRNA-p21, the expression of p21 decreased and, although hnRNP-K was present, the expression of the polycomb target genes was downregulated and the chromatin state of the genes was altered.

### 5.5. Circular RNAs in Colorectal Cancer and Their Role as miRNA Sponges

Circular RNAs (circRNAs) were reported more than 30 years ago by electron microscopy [289] and later on as several abnormally spliced transcripts in which exons from a candidate tumor suppressor gene* (DCC)* were scrambled during the splicing process in vivo [290]. However, they were considered as rare molecules that resulted from splicing artefacts or gene rearrangements. CircRNAs were rediscovered from RNA sequencing (2012/2013) and recent studies reporting high abundance, great diversity, and tissue and development specific expression indicate that they are not just a result of splicing errors. These studies have discovered thousands of endogenous circRNAs in mammalian cells, among them more than 2,000 circRNA in humans.

circRNAs are RNA molecules that form covalently closed continuous loops with joined 3′ and 5′ ends. They mainly arise from protein coding genes, from exons (exonic circRNA) or introns (intronic circRNA), by models of exon self-circularization, by lariat-driven circularization, or by back-splice events [291, 292]. In this last process, the downstream exons (3′ splice donor site) are spliced to upstream exons (5′ splice acceptor site) in the primary transcript, yielding a circular transcript. Reverse complementary sequences or RNA-binding proteins are necessary for circRNA biogenesis. For intronic lariat-originated circRNAs, a consensus motif containing a seven-nucleotide GU-rich element at the 5′ splice site and an eleven-nucleotide C-rich element upstream the branchpoint has been reported [292].

A characteristic of circRNAs is their stability and resistance to RNase R. They are related to different cancers, including CRC, where they exhibit an aberrant expression. Bachmayr-Heyda and coworkers [293] have reported that the ratio of circular to linear RNA isoforms was always lower in tumor compared to normal colon samples and even lower in CRC cell lines. A negative correlation between this ratio and the proliferation index, which could infer that circRNAs accumulate in nonproliferating cells, was described. They also predicted more than 1,800 circRNAs in human normal colon mucosa and tumor samples of CRC patients, a number which is in line with other reports about circRNAs.

RNA-seq analyses revealed that circRNAs were enriched in exosomes compared to the producer cells and more than 1,000 circRNAs were identified in human serum exosomes. Exosomes contain a specific cargo of protein, mRNA, and miRNA species, which can modulate recipient cell behaviors and may be used as biomarkers for diagnosis of human diseases. Li and coworkers described for the first time the presence of abundant circRNAs in exosomes and, interestingly, revealed that serum exosomal circRNAs were able to distinguish CRC patients from healthy controls. Compared to healthy subjects, 67 circRNAs were absent and 257 new circRNA species were detected in CRC patients [294].

circRNAs have been proposed as potential biomarkers for cancer diagnosis and targeted therapy [295, 296]. Furthermore, circRNAs associate with cancer-related miRNAs acting as miRNA sponges (Figure 3), binding to RNA-associated proteins to form RNA-protein complexes, and then regulating gene transcription [297]. Memczak and coworkers [296] found that a human circRNA, antisense to the cerebellar degeneration-related protein 1 transcript (CDR1as, renamed ciRS-7: circular RNA sponge for miR-7) was densely bound by miRNA effector complexes and harbors more than 63 conserved binding sites for miR-7 and is densely bound by AGO protein but not by unrelated proteins. Thus, ciRS-7 impairs the regulatory effect of miR-7 that, as we have previously described, is a tumor suppressor which is downregulated in a variety of cancers, among them CRC. As miR-7 modulates the expression of several oncogenes, ciRS-7/miR-7 interaction may play important roles in cancer-related pathways. CiRS-7 can be cleaved by miR-671 and its associated Argonaute protein, whereas it is not cleaved by miR-7 and Argonaute 2 [298, 299]. The second cirRNA highly expressed in murine testes, SRY, also functions as a miR-138 sponge; the circular SRY transcript has 16 binding sites for miR-138 and coprecipitates with AGO2 [300]. Although some of the molecular and biological roles played by circRNAs have been characterized, the role of cirRNAs as new star of noncoding RNA needs to be clearly established, the biological function of some of them remains largely unknown, and very few circRNAs have been described with the potential to act as RNA sponges.

Recently, a new type of circRNAs has been described arising from transcribed exons of distinct genes affected by chromosomal translocations and that encode oncogenic fusion proteins involved in tumorigenesis. They are called fusion circRNAs and can coexist with the oncogenic fusion proteins contributing to cellular transformation, promoting cell viability and resistance upon therapy, and have tumor-promoting properties in in vivo models [301]. The presence and effects of these f-circRNAs has been shown in acute promyelocytic leukemia (f-circPR and f-circM9 arising from gene fusions* PML/RARA* and* MLL/AF9*, resp.), in Ewing sarcoma (f-circEF1 from* EWSR1/FLI1* fusion), and in lung cancer (f-circEA1 from* EML4/ALK1* fusion).

## 6. Colorectal Cancer Treatments and Biomarkers

### 6.1. Classical Therapies and Response Biomarkers in Colorectal Cancer

CRC cancer can be triggered by alteration of a plethora of molecular mechanisms, whose accumulation ultimately leads to malignant transformation. As previously mentioned, the most frequent alterations reported in the onset of CRC are as follows: oncogene activation, tumor suppressor gene inactivation, mutations in the mismatch repair (MMR) enzymes, microsatellite instability (MIN), chromosomal instability (CIN), or epigenetic alterations. Chemotherapy treatments are directed towards the reversion of some of the effects caused by these alterations, but their efficacy is highly variable and requires the development of new adjuvant therapies against different targets. In addition, the availability of new biomarkers for CRC will greatly improve the efficacy of chemotherapy.

Initial standard drugs for CRC chemotherapy (see Table 3) were 5-fluorouracil (5-FU), leucovorin, or their combination (FL); these treatments offered an overall survival of about 8 to 12 months [302]. The introduction of combined therapies with irinotecan and/or oxaliplatin (FOLFOX, FOLFIRI, CapeOX, and FOLFIRINOX) showed significant improvement in disease-free survival and overall survival [303]. A further step was achieved with the use of monoclonal antibodies targeting EGFR or the vascular endothelial growth factor receptor (VEGFR) in combination with the aforementioned chemotherapeutic agents for the treatment of metastatic CRC [304–307]. In fact, the use of these antibodies is now recommended as the standard first-line chemotherapy in this type of tumors.

The monoclonal antibodies cetuximab and panitumumab, directed against EGFR, inhibit cell proliferation and tumor growth by binding to the extracellular domain of the receptor blocking the ligand-induced phosphorylation of the cytoplasmic domain and, consequently, downstream signaling (Table 3) [308]. In this context, it is important to point out that gene mutations in proteins downstream EGFR may be responsible for cetuximab or panitumumab resistance even in combined treatments with chemotherapeutic drugs.* KRAS* and* NRAS* have been extensively studied regarding resistance to chemotherapy regimens that include these monoclonal antibodies. In fact, in patients with* RAS* mutations in exons 2 (codons 12 and 13), 3, or 4, leading to constitutive activation of the protein, no beneficial effects are obtained with the monoclonal antibody treatments compared to standard chemotherapeutic regimens; only patients with wild-type* KRAS* or* NRAS* present a significant enhancement in survival [306, 307, 309–312]. Mutations of other molecular components of the signaling pathway downstream of EGFR (Ras-Raf-MEK-MAPKs) have also been evaluated as predictive markers for the anti-EGFR therapy. On this idea,* BRAF* mutations seem to be an indicator of poor prognosis even in wild-type* KRAS*, but results are still unclear [307, 309, 313, 314].

Antiangiogenic combined therapies are also frequently used for the treatment of metastatic CRC (Table 3). The humanized monoclonal antibody bevacizumab is directed against vascular endothelial growth factor A (VEGF-A), blocking in this way its binding to VEGFR1 and VEGFR2. This growth factor is proangiogenic and has been associated with tumor vascularization, progression, and metastasis, as well as providing protection against the cytotoxic effects of chemotherapy drugs. Bevacizumab blocks neovascularization of the tumor and improves the effectiveness of antitumoral drugs [315]. Patient response to bevacizumab was thought to be directly related to level of circulating VEGF-A as several studies linked high VEGF levels to a poor clinical outcome. However, the use of VEGF levels as predicted biomarker for treatment with bevacizumab is not currently advised as inconsistent data have been found. This may arise from the fact that several other molecules are involved in angiogenesis, and some of them are been analyzed as potential biomarkers for bevacizumab response [316–318].

Other approaches against the angiogenic pathway include the combined use of chemotherapic regimens with ziv-aflibercept or with ramucirumab. Ziv-aflibercept is a humanized fusion protein consisting of VEGF-binding portions from the extracellular domains of human VEGFR1 and VEGFR2, which are fused to the Fc portion of the human IgG1 immunoglobulin. It binds and inhibits VEGF-A, VEGF-B, and placental growth factor (PlGF), blocking in this way angiogenesis and decreasing vascular permeability. The combined treatment of metastatic CRC with FOLFIRI with ziv-aflibercept has shown a significant enhancement in overall survival [319]. Ramucirumab is a human monoclonal antibody against the extracellular domain of VEGFR2, interfering with VEGF-A binding and further angiogenic signaling. Its use combined with FOLFOX or FOLFIRI has also shown promising results for the treatment of patients with metastatic CRC [320–322].

The multikinase inhibitor regorafenib was approved in 2012 by the US Food and Drug Administration for the treatment of patients with metastatic CRC who have been previously treated with fluoropyrimidine-, oxaliplatin-, and irinotecan-based chemotherapy, an anti-VEGF therapy, and an anti-EGFR therapy (the latter only in those cases with wild-type* KRAS*). Regorafenib and its active metabolites inhibit multiple membrane-bound and intracellular kinases involved in normal cellular functions and in pathologic processes (as angiogenesis or tumor growth), including those in the RET, VEGFR1, VEGFR2, VEGFR3, KIT, PDGFR-*α*, PDGFR-*β*, FGFR1, FGFR2, TIE2, DDR2, Trk2A, Eph2A, RAF-1, BRAF, BRAFV600E, SAPK2, PTK5, and Abl pathways [323, 324].

Other biomarkers have been analyzed in order to check the response to monoclonal antibody treatments, mainly centered in anti-EGFR therapy. Besides* KRAS* and* NRAS*, mutations in* PIK3CA*,* TP53*,* PTEN*, and* EGFR* have been proposed as predictive biomarkers with variable success. Regarding mutations in* PIK3CA* (which encodes the PI3K catalytic subunit), conflicting results have been obtained [325]. It has been proposed that anti-EGFR monoclonal antibody treatments might be efficient in tumors where p53 is inactivated [326], whereas PTEN loss correlates with lack of response to cetuximab and panitumumab [325, 327].

### 6.2. miRNAs as Predictive Biomarkers and Therapeutic Targets in CRC

As described above, one of the major problems for the management of CRC is the inherent or acquired resistance to therapeutic treatments. As miRNAs are important regulators of cell signaling pathways involved in carcinogenesis, progression, invasion, angiogenesis, and metastases in CRC, they are being analyzed as potential predictive and prognostic factors, or even as therapeutic targets themselves. In fact, the involvement of the dysregulation of several miRNAs has been linked to tumor progression and response to anticancer therapies [144, 328–330]. miRNAs are also required for normal immune system development and function and aberrant expression of miRNAs has been observed in various tumor types leading to immune disorders or immune evasion. Thus, miRNAs could also be considered as potential targets in the regulation of the immune response in CRC in order to develop new therapeutic strategies [331].

miRNAs are very stable molecules that resist prolonged storage, exposure to high or low pH values, or even boiling and can be detected in archival tissue specimens and serum. These characteristics are quite interesting for their use as biomarkers as they can be extracted for analysis from blood, plasma, serum, and various body fluids and in frozen or formalin-fixed paraffin-embedded tissues [332, 333]. The discovery that extracellular miRNAs circulate in the bloodstream and that such circulating miRNAs are quite stable raised the possibility that miRNAs may be probed in the circulation and can also serve as novel diagnostic makers. These molecules are protected from degradation by their inclusion in lipid vesicles or by their interaction with plasma proteins, as was originally shown by El-Hefnawy and coworkers [334]. Depending on their size and mode of release from cells, miRNAs can be included into exosomes, microvesicles, or apoptotic bodies. Exosomes (50–100 nm in diameter) originate from the endosome and are released from cells when multivesicular bodies fuse with the plasma membrane; microvesicles are released from the cell through blebbing of the plasma membrane. miRNAs are also found in circulation in microparticle-free form, associated with high-density lipoproteins (HDL) or bound to RNA-binding proteins such as Ago2 or NPM1 (nucleophosmin). It is not known how these miRNA-protein complexes are released from the cell; they may be released passively, as byproducts of dead cells, or actively, in a miRNA-specific manner, through interaction with specific membrane channels or proteins [335].

Cells can select some miRNAs for cellular release while others are mainly retained within the cell. Pigati and coworkers analyzed normal and malignant epithelial cells and reported that 66% of the miRNAs were released in quantities that reflected their intracellular level and 13% were selectively retained in the cell with very low secretion, but 21% of the miRNAs were actively secreted [336]. They also observed that pre-miRNAs were also secreted together with mature miRNAs. In fact, some cells secrete preferentially the precursor forms rather than the mature ones, as reported for mesenchymal stem cells [337]. The release of miRNA-containing exosomes seems to be ceramide dependent; human CRC cells release miRNAs in ceramide-rich exosomes, and inhibition of enzymes involved in ceramide biosynthesis (as sphingomyelin phosphodiesterase 3) strongly decreases the secretion of miRNAs into exosomes [338].

#### 6.2.1. miRNAs as Diagnostic and Prognostic Markers

miRNAs are beginning to be used as diagnostic markers of CRC [21]. Serum miR-21 and miR-92a have been analyzed as biomarkers in the diagnosis and prognosis of CRC [339]. Patients with advanced adenoma or with CRC showed significantly higher levels compared to healthy controls. They have been also studied in stool, but they showed lower sensitivity and specificity than in serum for the detection of CRC [340]. Elevated serum levels of miR-92a correlate well with CRC and poor survival [339], whereas miR-21 is not specific to CRC but is also found increased in the plasma of patients with other cancer types [341]. In another study, high levels of circulating miR-34a and low miR-150 levels distinguished groups of patients with polyps from those with advanced CRC, and low circulating miR-150 levels separated patients with adenomas from those with advanced cancer [342]. mir-1290 has been also described as a novel biomarker for early detection, recurrence, and prognosis in human CRC [343]

In order to achieve a more precise screening for CRC, panels of plasma miRNAs have been devised: a panel consisting of eight miRNAs (miR-532-3p, miR-331, miR-195, miR-17, miR-142-3p, miR-15b, miR-532, and miR-652) could identify polyps from controls, and a panel of three miRNAs (miR-431, miR-15b, and miR-139-3p) distinguished stage IV CRC from controls [344]. miR-135b has been also found to be increased in CRC and adenomas compared to normal adjacent tissue, as well as in stool specimens where levels of this miRNA increased in patients with adenomas compared to those with CRC and were higher than in control patients or patients with inflammatory bowel disease. Moreover, miR-135b levels in stool decreased significantly after surgery, suggesting that this miRNA may act as a biomarker for early-stage CRC [345]. Fecal miR-106a has been also proposed as a useful marker for colorectal cancer patients with false-negative results in immunochemical fecal occult blood test [346].

Finally, Ahmed [347] performed a global analysis of miRNA expression in stool from 12 patients with CRC (3 per stage; 0-I, II, III, and IV) and 3 controls and found 141 miRNAs overexpressed in CRC and 61 with reduced expression. After selecting 20 miRNAs, they carried out an additional study using modified real-time quantitative PCR with stool samples from 60 patients (20 per group) and found 12 miRNAs overexpressed in CRC and whose expression increased with the stage (miR-7, miR-17, miR-20a, miR-21, miR-92a, miR-96, miR-106a, miR-134, miR-183, miR-196a, miR-199a-3p, and miR-214). Conversely, they also found 8 miRNAs that showed reduced expression in CRC and that decreased with stage progression (miR-9, miR-29b, miR-127-5p, miR-138, miR-143, miR-146a, miR-222, and miR-938). Taking into account these finding, the authors propose that a chip can be developed to facilitate diagnosis of CRC from stool or blood samples.

In addition to diagnosis, miRNAs are beginning to be considered as promising predictive markers for response to therapy. On this idea, miR-31-3p and -5p were identified as good markers of response to cetuximab treatment in wild-type* RAS* patients with metastatic CRC as nonresponders presented significantly higher expression levels of these two miRNAs compared to responders. However, no association between time to progression and the expression of these miRNAs was found in patients treated with panitumumab [216]. Similar associations have been found for other miRNAs and treatments. For example, miR-143 low-level expression in primary tumors was found as a good predictive factor for the effectiveness of capecitabine treatment as first-line monotherapy in patients with metastatic CRC [348]. miR-320e has been also identified in two clinical trials cohorts as a novel prognostic biomarker that is associated with adverse clinical outcome in stage III CRC patients treated with 5-FU-based adjuvant chemotherapy (FOLFOX) [349]. These findings have important implications for the personalized management of CRC patients.

The presence of SNPs in specific miRNA has also been associated with prognosis and response to chemotherapy treatments in CRC. The expression of specific miR-492 variants in CRC patients has been reported in association with a better prognosis and progression-free survival [350]. Regarding response to therapy, several articles describe that* KRAS-LCS6* variant is of particular interest due to an alteration in the binding site of let-7 miRNA at the 3′UTR of K-Ras. Graziano and coworkers have reported that patients with metastatic CRC without mutations in* BRAF* that received anti-EGFR therapy (cetuximab plus irinotecan) showed a much lower survival if they presented the* KRAS-LCS6* SNP than patients without it [351]. However, apparently opposite results were obtained by Zhang and coworkers in patients with wild-type* KRAS* treated only with cetuximab and no irinotecan [219]. Thus, care must be taken when considering this SNP as a predictive response parameter as it strongly depends on the chemotherapy regimen and in the mutational status of* KRAS* and* BRAF*.

#### 6.2.2. miRNAs Involved in Chemoresistance and Radioresistance

Chemotherapy sensitivity or resistance is strongly dependent on protein targets that are epigenetically modified that involve variations in the intracellular expression of miRNAs and lncRNAs. The literature regarding the involvement of miRNAs in the acquisition of resistance in CRC cells to conventional chemotherapy agents such as 5-FU, oxaliplatin, or irinotecan is ample (Table 4). Most of the findings rely on in vitro experiments with cultured cells and some of them are even controversial. For example, cells resistant to 5-FU treatment have been reported to have increased expression of miR-192 and miR-215, whose target is thymidylate synthase (TYMS), a potential target of 5-FU. Surprisingly, however, these miRNAs seemed to increase resistance to 5-FU, rather than improving the drug's efficacy [186]. On the other hand, miR-129 or miR-203, whose mRNA target is also TYMS, behaves in a completely opposite manner; they are downregulated in 5-FU resistant cells, but their restoration induces chemosensitivity to this agent [196, 208]. It is also somehow controversial the fact that let-7g overexpression, that belongs to a miRNA family normally described as tumor suppressors, was associated with chemoresistance to S-1 (composed of 5-FU prodrug tegafur, a dihydropyrimidine dehydrogenase inhibitor, gimestat, and an inhibitor of orotate phosphoribosyltransferase, otastat potassium, in a molar ratio of 1 : 0.4 : 1) in colon cancer [177].

In general, those miRNAs whose expression is upregulated in chemoresistant cells repress the expression of targets related directly or indirectly to the induction of apoptosis or to the control of proliferation. An example of the former is miR-153, which is upregulated in resistance to oxaliplatin in vitro and in vivo. This miRNA represses the expression of the Forkhead transcription factor FOXO3a repressing in this way apoptosis through reduced caspase-3 activation, upregulation of antiapoptotic genes, and downregulation of proapoptotic ones as* PUMA* and* Bim* [205]. Regarding dysregulation of the cell cycle, it has been proposed that miR-520g may confer chemoresistance to 5-FU and oxaliplatin in CRC cells through downregulation of p21 expression. In addition, p53 inhibits miR-520g expression and the loss of p53 function causes an increase in the expression levels of miR-520g, suggesting an important role of this miRNA as a potential target for therapy to overcome drug resistance in CRC patients [189].

miRNAs have also an impact on the resistance of CRC to targeted chemotherapy or combined regimens (Table 4). The overexpression of several miRNAs has been directly related to resistance to monoclonal antibody-based therapies. As previously discussed, miR-31 is upregulated in metastatic CRC and can be used as a predictive marker of the efficacy to cetuximab treatment but not to panitumumab. However, it is yet unclear the mechanism by which this miRNA enhances resistance [216]. Upregulation of miR-199a-5p and miR-375 induces cetuximab resistance in CRC cells by targeting the tumor suppressor* PHLPP1*, a negative regulator of the Akt pathway [218]. Ragusa and coworkers correlated high expression levels of miR-146b-3p and miR-486-5p with resistance to cetuximab in patients with constitutive activation of* KRAS* signaling, suggesting their involvement in EGFR pathway [215]. In the same study, they describe that upregulation of members of the let-7 family may enhance sensitivity of metastatic CRC to anti-EGFR treatments by targeting K-Ras mRNA. In a similar way, Suto and coworkers showed that miR-7 induced sensitivity to cetuximab in CRC cell lines by targeting EGFR and RAF1 mRNAs and suppressing ERK1/2 and phospho-Akt expression [136]. miR-126 was described as a potential tumor suppressor involved in regulation of angiogenesis and thus was suspected to be involved in the outcome of bevacizumab treatment. However, although high expression levels of miR126 in primary CRC tumors were detected in XELOX responsive patients [352], high levels of circulating miRNA-126 were associated with bevacizumab plus XELOX resistance [217].

miRNA deregulation in CRC tissues may also influence the activity of signaling pathways involved in the response to combined chemo- and radiotherapy of these tumors. Colorectal cell lines exposed to continuous low-dose radiation overexpress miR-622, and this overexpression is maintained in surviving cells. Resistance arises from a downregulation of Rb, whose mRNA is a direct target of miR-622. In fact, overexpression of Rb in these cells reverses radioresistance [353]. The opposite effect has been detected for miR-630 and miR-106b, whose upregulation is directly correlated to radiosensitivity by targeting BCL2L2 and TP53RK mRNAs (miR-630), both involved in cell survival and apoptosis inhibition [354], or PTEN and p21 (miR-106b), leading to the activation of Akt which promotes cell survival and proliferation [355]. Long noncoding RNAs are also involved in radiosensitivity. The long intergenic lincRNA-p21 has been shown to increase the sensitivity of radiotherapy for human CRC by targeting the Wnt/*β*-catenin signaling pathway [287].

#### 6.2.3. miRNA as Therapeutic Targets and Tools in CRC

As previously discussed, the expression of miRNAs is altered in CRC; some miRNAs may act as oncogenes (oncomiRs) and others as tumor suppressor genes. It is feasible to manipulate their expression by injecting miRNAs in a similar way to the use of antisense mRNAs or RNAi, blocking the activity of oncomiRs or well replacing tumor suppressor miRNAs to restore loss of function. In any case, before applying these technologies, it is essential to establish first which miRNAs are up- and downregulated in CRC, followed by loss/gain studies in vitro or in animal models. After identifying potential miRNA targets, a pharmacological analysis must be carried out with in vivo miRNA delivery studies and analysis of pharmacokinetic and pharmacodynamics. Finally, clinical trials should be carried out to evaluate efficacy and safety of the potential treatments [356].

Mature miRNAs can be inhibited using different tools: antisense oligonucleotides (anti-miRs, antagomirs, or ASOs), miRNA sponges, miRNA masking, anti-miR peptides, or small molecule inhibitors. A miRNA sponge is a transcript (mRNA) expressed from plasmids possessing multiple tandem binding sites for targeted miRNAs that block selectively a whole family of related miRNAs. For example, in breast cancer cells, Jung and coworkers demonstrated that a multipotent miRNA sponge against miR-21, miR-155, miR-221, and miR-222 strongly inhibited cell migration in a much stronger way than single miRNA targeting [357]. Sponges have been widely used to investigate miRNA function in vitro, but their utility in vivo has been limited to transgenic animals in which the sponge mRNA is overexpressed in target tissues [358]. Interestingly, it seems that some large and circular noncoding RNAs as well as pseudogene transcripts could serve as natural sponges to regulate cellular miRNA availability and lead to upregulation of downstream target genes [261, 359].

The miRNA masking technology was developed by Choi and coworkers [360] and consists of single-stranded 2′-O-methyl-oligonucleotides complementary to miRNA binding sites in the 3′UTR of target mRNAs that disrupt the interaction of specific miRNA-mRNA pairs. An advantage of this method is that avoids potential off-target secondary effects.

Approaches using small molecule inhibitors to manipulate miRNA expression and function are also being developed. Screening of this type of compounds has identified small molecules that can specifically inhibit the expression of miRNAs, as azobenzene, which affects miR-21 expression [361], or several molecules that inhibit miR-122 that could be useful for HCV therapy [362]. These molecules do not inhibit target recognition by the miRNAs but rather modulate the transcription of targeted miRNAs. However, these molecules are still of limited therapeutic potential due to their high EC_50_ values and the lack of specific inhibitors for many miRNAs. On the other hand, some fluoroquinolone antibiotics, as enoxacin, have been described to enhance the effect of siRNAs or miRNAs by increasing the binding affinity of TRBP to the miRNA precursors. It does not present specificity for miRNA sequences but induces a global increased expression of miRNAs that could be interesting for tumors where this expression is lowered [363].

**Figure 5:**
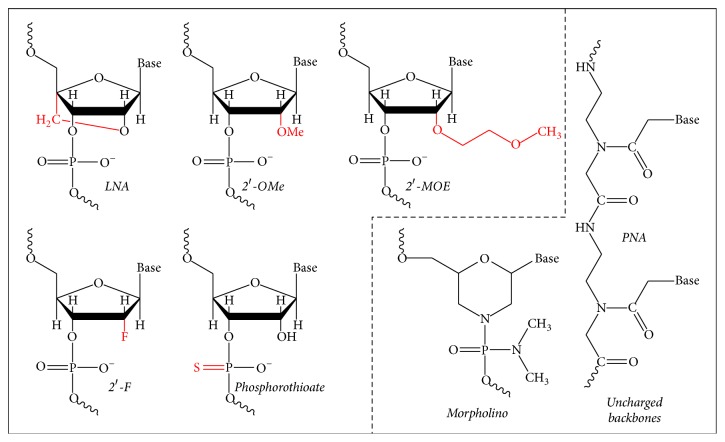
Chemical modifications of miRNA-targeting modulators. Anti-miR oligonucleotides have been chemically modified in order to achieve better stability against serum nucleases and to increase the binding affinity to targeted miRNAs. Most of the modifications are at the 2′ position of the sugar moiety, as 2′-*O*-methyl (2′-OMe), 2′-*O*-methoxyethyl (2′-MOE), and 2′-fluoro (2′-F). Locked nucleic acid (LNA) is a bicyclic RNA analogue in which ribose is locked by introduction of a methylene bridge between the 2′ oxygen and the 4′ carbon of the pentose. In addition, most anti-miR oligonucleotides contain phosphorothioate backbone linkages in which sulfur replaces one of the nonbridging oxygen atoms in the phosphate group. Morpholino oligomers replace the ribose with a methylenemorpholine ring (to which bases are attached) with phosphorodiamidate linkages. PNA oligomers are oligonucleotide analogues in which the ribose-phosphate backbone has been replaced with a peptide-like structure containing* N*-(2-aminoethyl)-glycine units. Both morpholino and PNA oligomers are uncharged, which facilitates the interaction with targeted miRNAs.

Antisense inhibition of mature miRNAs is nowadays still the technology which has received more attention, mainly regarding anti-miRs or ASOs which specifically target oncomiRs. These anti-miRs bind to the miRISC complexes blocking the interaction of miRNAs with their endogenous mRNA targets. This approach has been tested in human colon carcinoma cells in culture and in mice models of CRC targeting overexpressed miRNAs. Specific silencing of miR-135b has been shown to effectively inhibit tumor proliferation in mice models and to induce apoptosis in SW480 human colon carcinoma cells [364] or reduce the migratory ability in HCT-116 cells [345]. In addition to miR-135b, other potential oncogenic miRNAs have been tested using this knockdown technology as miR-20a, miR-21, miR-31, miR-95, or miR-675 in other human colorectal carcinoma cell lines [124, 180, 182, 276, 365]. A potential drawback of this technology is that unmodified RNA oligonucleotides are quite sensitive to serum nucleases for their potential in vivo administration. In addition, they cannot penetrate cell membranes without being modified or encapsulated to enable their entry into the cell cytoplasm. For these reasons, several lines of research are directed towards increasing resistance of anti-miRs to nucleases by chemical modification, to enhance their binding affinity to mRNA targets and to improve their pharmacokinetics and pharmacodynamics in vivo (Figure 5). The 2′-*O*-methyl (2′-OMe) modification was one of the first attempts (reported in 2004) to successfully block miRISCs in* Drosophila melanogaster* and in human cultured cells. However, although these 2′-OMe modified oligonucleotides were more effective, they were still susceptible to degradation by serum nucleases. To increase resistance, oxygens in the phosphate backbone were replaced with sulfur atoms to form phosphorothioate-modified miRNAs. These modifications showed also improved pharmacokinetics [366]. Further improvement of anti-miR showed that modifications were more efficient in the 3′ end and that linkage of cholesterol to this end also increased the pharmacology and efficacy of specific anti-miRs [367]. Other modifications at the 2′ sugar position have been proposed to increase the efficacy of anti-miRs, such as 2′-*O*-methyoxyethyl (2′-MOE), 2′-fluoro (2′-F), and locked nucleic acid (LNA; bicyclic nucleic acid with a methylene bridge between 2′ oxygen and the 4′ carbon) modifications (for review see [329, 368]). Among these modifications, LNA has shown an exceptional binding affinity to miRNAs, which has made it possible to achieve efficient inhibitions with rather short sequences that bind only the seed regions of the target miRNAs [369].

Anti-miR peptides are artificial peptides (peptide nucleic acids or PNAs) that behave as uncharged DNA or RNA analogues in which the sugar backbone has been replaced with a peptide-like backbone which consists of* N*-(2-aminoethyl)glycine units to which the nucleobases are attached. These PNAs bind to target miRNA more tightly than equivalent oligonucleotides, are more stable, and can be administered systemically with low toxicity. Similarly, morpholinos are uncharged oligonucleotide analogues with a slightly increased binding affinity to complementary miRNAs [329, 368].

Other approach for miRNA therapeutics deals with the recovery of the activity of tumor suppressor miRNAs that are downregulated or not expressed at all in CRC or other types of cancer. For this purpose, current research is using synthetic RNA duplexes that, as described above for miRNA inhibition, harbor chemical modifications to improve stability, nuclease resistance, and cellular uptake [368, 370]. These are referred to as miRNA mimics and contain one strand identical to the miRNA of interest (guide or antisense), whereas the opposite strand (passenger or sense) is normally less stable. This passenger strand has been linked in some studies to a molecule as cholesterol or peptides to enhance cellular uptake. In addition, chemical modifications are normally introduced to avoid loading of this strand into the RISC complex, as 5′-*O*-methylation, but still allowing its degradation after separation of the antisense strand [371]. The antisense strand has to be loaded into the RISC complex and thus, its modifications for stability have to be carried out with more care to avoid interference with the formation of the complex. This therapeutic approach has been tried in colon and hepatocellular carcinomas and hepatic metastasis (that may arise from primary CRC) and, in cell culture and in mice orthotopic models, recovers the expression of downregulated tumor suppressor miRNAs miR-26a, miR-33a, miR-34a, or miR-145. Overexpression of these miRNAs with mimics resulted in inhibition of cancer cell proliferation and apoptosis, or in tumor reduction, prolonged survival, and disease protection in animals [356, 372–374]. In 2013, the first miRNA mimic entered phase 1 trial in patients with primary liver cancer or metastatic cancer with liver involvement under the name of MRX34, a double-stranded RNA delivered by Smarticles (licensed liposomes which comprise different mixtures of palmitoyl oleoyl phosphatidyl choline, dioleoyloxytrimethylammoniumpropane, 1,2-dimyristoylglycerol-3-hemisuccinate, and cholesterol) (http://www.mirnatherapeutics.com/) [113]. However, multiple immune-related severe adverse events were observed in patients dosed with MRX34 over the course of the trial and the company decided to halt the study.

Restoration of tumor suppressor miRNAs still presents several challenges. Treatment with the synthetic RNA duplexes may recover the expression of downregulated miRNAs, but there is always a risk of overexpressing them or that cells that normally do not express the targeted miRNAs suffer off-target effects. These potential unwanted side effects of miRNA mimics reveal the importance of improving the targeted delivery to the appropriate cells or tissues, also quite important when silencing oncomiRs.

As discussed above, the use of miRNA therapeutics in vivo presents considerably more challenges than research in vitro. Much progress has been made regarding the resistance of therapeutic miRNAs to nucleases or to increase the binding affinity of anti-miRs or miRNA mimics to mRNA targets in the miRISC complexes. However, the main challenge for development of miRNA-based therapeutics is the design of efficient and safe methods for the delivery in vivo of miRNAs to targeted tissues or cells [329, 368]. Research has been mainly centered in delivery of siRNAs, but these advances can be readily applied to miRNA mimics and anti-miRs.

Conjugation of cholesterol to the 3′ end of miRNAs was one of the first attempts; it allowed incorporation of siRNAs or miRNA mimics into HDL and LDL lipoproteins [375]. Since them, several other molecules have been checked, as *α*-tocopherol linked to the 5′ end of the antisense strand, receptor-targeting ligands for cell binding (i.e., CpG-containing oligonucleotides directed for cells expressing TLR9 receptor), or cell-penetrating peptides for crossing cell-surface or endosomal membranes, as pHLIP. This peptide is a 38-amino-acid-long hydrophobic, negatively charged peptide that, under slightly acidic conditions (around pH 6.5), inserts as a helix into cell membranes. This peptide was unsuccessful when used with oligonucleotides but worked efficiently with PNAs linked via a disulfide bond [376].

Lentivirus- or adeno-associated virus-based miRNA expression constructs have been also studied for the delivery of tumor suppressor miRNAs, and some of these constructs are being used in several clinical trials for gene therapy. Liposome-based methods have been also widely tested for the encapsulation of siRNAs and miRNAs or analogues. These liposomes can even be coated with carbohydrates, peptides, or antibodies to direct them towards cell types that express specific receptors or antigens. Similarly, siRNAs or miRNAs can be incorporated in polymer-based nanoparticles (10–100 nm), as those containing polyethyleneimine that have been reported to be efficient for the therapy of CRC in cell culture and mouse xenografts by restoring miR-33a or miR-145 function [373, 374]. Antibody-based methods have been also described. The most common approach is to design chimeric proteins consisting of an RNA-binding protein (as protamine or synthetic positively charged peptides) fused to Fab or single-chain variable fragments from antibodies directed towards antigens expressed in targeted cells.

Several challenges remain to be addressed before miRNA therapeutics can reach safely the clinic. A first one is associated with the fact that, from the 2588 mature miRNAs described so far in humans (miRBase 21), only around 200 are sufficiently expressed to be feasible targets for therapy [329]. Many of these miRNAs belong to families with similar seed regions and thus, it is quite normal that, for example, an anti-miR directed against one member of one of these families may affect also other miRNAs with similar or identical seed region under physiological conditions. A second important aspect to be considered is that miRNA mimics or anti-miRs can elicit an immunological response mainly through the innate immune system, whose cells express Toll-like receptors (TLRs) [377]. These receptors recognize dsRNAs (TLR3) or single-stranded GU-rich RNAs (TLR7 and TLR8) and evoke an interferon-*α* response by plasmacytoid dendritic cells [378]. This response can be reduced by chemical modification of the sense strand; on this idea, Hornung and coworkers observed that LNA modifications strongly reduced the immunostimulatory effects of siRNAs [378]. Although these modifications may be efficient in order to get nuclease resistance or stability of miRNAs, some of them may elicit other undesirable responses, affecting, for example, blood coagulation, activation of the complement cascade, the immune system (phosphorothioate-containing oligonucleotides), or liver toxicity (i.e., LNA-modified anti-miRs) [329]. Finally, much care has to be taken when administering anti-miRs in vivo as blocking the function of certain oncomiRs may negatively affect physiological responses where those miRs exert specific functions. Thus, cell or tissue targeting is essential to avoid these potential problems. In addition, it is also difficult to ensure whether the appropriate dose of miRNA reaches targeted cells and becomes incorporated into RISCs. Some of these potential problems could be addressed with more efficient delivery systems in order to direct miRNAs towards specific cells or tissues, facilitate intracellular transport, and further release into the cytoplasm. This could allow decreasing the therapeutic dose of miRNA mimic or anti-miR in vivo minimizing adverse effects.

## Figures and Tables

**Table 1:** Hallmarks in miRNA discovery and its relationship to cancer.

Year	Hallmark
1993	*The first regulatory noncoding RNA was identified: lin-4* Two discoveries identified a novel mechanism of posttranscriptional gene regulation. The first miRNA lin-4 was described in 1993 as asmall temporal RNA (stRNA) in the laboratory of Ambros working with the nematode *Caenorhabditis elegans*. In this system, the transition from the first to the second larval stage fates requires the 22-nucleotide lin-4 RNA. The gene *lin-4* encodes a small RNA, which is a non-protein-coding regulatory RNA molecule. In the same issue of cell, the group of Ruvkun reported the first miRNA target gene, the heterochronic gene lin-14, that is regulated by lin-4 mediating the temporal pattern formation in *C. elegans*. The sequence of lin-4 has antisense complementarity to lin-14 mRNA that encodes lin-14 protein [80, 81].

2000	*The second small temporal RNA and the first in humans was identified: let-7* Seven years later the second regulatory stRNA, let-7, was discovered. The transition from late larval to adult cell fates in *C. elegans* requires the 21-nucleotide let-7 RNA. This stRNA negatively regulates, among others, lin-14 and lin-28 through RNA-RNA interactions with their 3′ untranslated regions. The sequential stage-specific expression of the lin-4 and let-7 RNAs regulates the timing of *C. elegans* development. The let-7 RNA showed its conservation across species, including *H. sapiens*. In humans, let-7 was detected at different expression levels in several tissues, including brain, heart, kidney, liver, lung, trachea, colon, small intestine, spleen, stomach, and thymus [82, 83].

2001	*The term miRNA is introduced* In addition to lin-4 and let-7, several stRNAs with regulatory functions were discovered using bioinformatics and cDNA cloning. Three papers published in the same issue of Science showed the existence of small RNAs involved in posttranscriptional regulation of target mRNA in vertebrates and invertebrates. These RNAs were named microRNAs (miRNA) to refer to this class of small regulatory RNAs [84–86].

2002	*miRNA is associated with cancer* The relevance of miRNAs to cancer was suggested by changes in their expression patterns and recurrent amplification and deletion of miRNA genes in tumors. The first report suggesting a role of miRNAs in cancer described a frequent 13q14 deletion that encoded the miRNA-15a/16-1 cluster reducing its expression in chronic lymphocytic leukemia. Both genes were deleted or downregulated in 68% of analyzed cases. Two years later, the same group found that a significant percentage of miRNAs is located at fragile sites and in genomic regions altered in cancers, including regions of amplification or loss of heterozygosity or breakpoints. They suggested that miRNAs were a new class of genes with a relevant role in human cancer pathogenesis [7, 58].

2003	*miRNAs in colorectal cancer* A total of 28 different miRNA sequences were identified in a colonic adenocarcinoma and normal mucosa. Among them, miR-21, miR-143, miR-145, and miR-200c were expressed. In colorectal cancer, two different miRNAs, miR-143 and miR-145 exhibited significantly reduced levels of the mature miRNAs compared to normal mucosa specimens. The maintenance of constant levels of unprocessed hairpin precursors in both normal and tumor tissues suggested that altered transcription is not responsible for reduced miRNA levels. Authors proposed that the reduction is due to posttranscriptional processes such as a reduced Dicer-processing activity in the neoplastic cells or reduced stability of these specific miRNAs [87].

2002-2003	*miRBase: the miRNA sequence database* miRBase was established in 2002 as a miRNA registry. The criteria for the identification of miRNAs was published in 2003. The miRBase grew from the miRNA registry resource set up by Griffiths-Jones in 2003 and is the public repository for all published miRNA sequences and annotation data. Its aim is assigning stable and consistent names to newly discovered miRNAs. The first release of miRBase in 2002 contained 218 miRNA loci from five species. Since then, the number of miRNAs discovered has increased exponentially. The miRBase is freely available at http://www.mirbase.org/ [60–62].

2004	*miRNAs as molecular biomarkers* let-7 expression is associated with survival of lung cancer patients. This was the first time that miRNAs are suggested as prognostic markers. The article described that let-7 expression was reduced in lung cancers and that lung cancer patients with low let-7 expression levels had a significantly shorter survival after potentially curative resection. Currently, the clinical utility of miRNAs as diagnostic/prognostic biomarkers has been demonstrated in several types of cancer by numerous studies using tumor samples [88].

2005	*Function of miRNAs in cancer* The first reports addressing the biological function of miRNAs in cancer were published. These articles described that miR-15 and miR-16, the first two miRNAs associated with cancer, play a role in apoptosis regulation by targeting the antiapoptotic bcl-2 mRNA. They also reported the first miRNA-target interaction with relevance to cancer: human Ras expression is regulated by let-7 in cell culture. In fact, let-7 expression is decreased in lung cancer compared with normal tissue, and it correlates with the increased Ras protein levels detected in lung tumor samples. Since then, hundreds of publications have reported on the role of miRNAs in tumors [89–91].

2005	*The expression of miRNAs is regulated by transcription factors* It is described that c-Myc activates the expression of a cluster of six miRNAs on human chromosome 13. In turn, the expression of a target of c-Myc, the transcription factor E2F1, is negatively regulated by two oncogenic miRNAs in this cluster, miR-17-5p and miR-20a [92].

2005–2007	*Role of miRNAs as candidate components of oncogene and tumor-suppressor networks* The role of miRNAs as oncogenes (oncomiRs) or tumor suppressors involved in a variety of pathways deregulated in cancer was reported. The polycistronic miRNA cluster miR-17-92, located in a region of DNA that is amplified in human B-cell lymphomas, is reported as a potential human oncogene. Other studies, using different types of tumors,also described the role of miR-143, miR-145, miR-372, miR-373, and miR-155/BIC as oncogenic miRNAs. Conversely, five independent reports describe that the miR-34 family of evolutionarily conserved miRNAs are directly induced by p53 in response to DNA damage and oncogenic stress. miR-34a was identified as a miRNA component of the p53 network, revealing an interplay between proteins and noncoding RNAs in a tumor-suppressor pathway [93–102].

2007	*miRNAs “sponges”* The initial term “target mimicry” was coined in plants to define the mechanism of inhibition of miRNA activity discovered studying the phosphate homeostasis in *Arabidopsis thaliana*. In the same year, there is a report in humans on specific competitive inhibitors from transcripts expressed from strong promoters that contain multiple tandem binding sites to several miRNA seed families; they were named “miRNA sponges” [103, 104].

2007–2009	*miRNAs and metastasis* miRNAs are also involved in metastasis; they can promote or inhibit metastasis. The first miRNA described as a metastasis activator was miR-10b, that positively regulates cell migration and invasion in vitro and is capable of initiating tumor invasion and metastasis in vivo. Expression levels of miR-10b in primary breast carcinomas correlate with clinical progression. Its expression is elevated in about 50% of metastatic breast tumors compared with metastasis-free tumors or normal breast tissues. Human miR-373 and miR-520c also stimulate cancer cell migration and invasion in vitro and in vivo. On the contrary, other miRNAs can prevent tumor metastasis. Breast cancer patients with low expression levels of miR-335, miR-126, and miR-206 had a shorter median time to metastatic relapse. Restoration of their expression in breast cancer cell lines decreased the number of metastases in inoculated mice [105–108].

2008	*Circulating miRNA biomarkers* miRNAs are detected in blood samples (plasma, platelets, erythrocytes, and nucleated blood cells). This pointed out that endogenous plasma miRNAs are protected in some manner to prevent their degradation. Due to their stability in the circulation, miRNAs began to be considered for their potential use as biomarkers for different pathologies [109–112].

2013	*miRNA therapeutics* miR-34a mimic (MRX34) enters Phase 1 clinical study in liver cancer and other solid tumors with liver involvement, as well as hematological malignancies. Regretfully, this study was halted in 2016 due to immune-related serious adverse events [113].

**Table 2:** Oncogenic and tumor suppressor miRNAs involved in colorectal cancer.

miRNA	Verified targets	Role in colorectal cancer	References
*Oncogenic miRNAs (upregulated)*			
miR-18a	ATM	Blocking apoptosis, DNA repair, and sensitivity to etoposide	[114]
miR-21	PTEN, PCDC4, TGFBR2, CDC25A	Proliferation, apoptosis, invasion, migration, CSC maintenance	[115–117]
miR-29a	KLF4	Invasion, metastasis	[118]
miR-31	HIF1A, RhoBTB1, RASA1	Proliferation, migration, invasion, tumor growth	[119–121]
miR-32	PTEN	Proliferation, migration, invasion, apoptosis	[122]
miR-92a	PTEN	Proliferation, migration, invasion, apoptosis, EMT	[123]
miR-95	SNX1	Proliferation, tumor growth	[124]
miR-96	TP53INP1, FOXO1, FOXO3A	Proliferation	[125]
miR-103	DICER, PTEN	Proliferation, migration, tumor growth	[126]
miR-135a/b	APC	Proliferation	[127]
miR-155	MLH1, MSH2, MSH6	Altering DNA damage response	[128]
miR-181a	WIF-1, PTEN	Proliferation, migration, invasion, tumor growth, liver metastasis, metabolic shift, EMT	[129, 130]
miR-182	FOXF2	Cell growth, invasion, increased *β*-catenin activity	[131]
miR-196b	FAS	Blocking apoptosis	[132]
miR-214	PTEN, PDLIM2	Inflammation	[133]
miR-223	RASA1	Tumor growth	[134]
miR-224	SMAD4	Metastasis	[135]

*Tumor suppressor miRNAs (downregulated)*			
let-7	KRAS	Proliferation	[91]
miR-7	EGFR, RAF1	Proliferation	[136]
miR-18a^*∗*^	KRAS	Proliferation	[137]
miR-26b	TAF12, PTP4A1, CHFR, ALS2CR2	Proliferation, apoptosis, invasion, migration	[138]
miR-27b	VEDFC	Proliferation, angiogenesis	[139]
miR-34a	SIRT1	Apoptosis	[140]
miR-100	RAP1B	Proliferation, invasion, apoptosis	[141]
miR-101	SPHK1	Angiogenesis	[142]
miR-124	STAT3	Proliferation, apoptosis, tumor growth, differentiation	[143]
miR-126	VEGFA, IRS-1, CXCR4	Proliferation, migration, invasion, cell cycle arrest, angiogenesis	[144–146]
miR-133a	FSCN1, LASP1	Proliferation, invasion, migration, tumor growth, metastasis, phosphorylation of ERK/MEK	[147, 148]
miR-133b	TBPL1, CXCR4	Proliferation, invasion, migration, apoptosis	[149, 150]
miR-139	IGF1R, NOTCH1	Proliferation, migration, invasion, apoptosis, tumor growth, cell cycle arrest	[151, 152]
miR-143	ERK5, KRAS, IGF1R	Proliferation	[153, 154]
miR-144	MTOR	Proliferation	[155]
miR-145	IRS1, NRAS, IGF1R	Proliferation, invasion, migration, angiogenesis, tumor growth, metastasis	[153, 154, 156]
miR-148b	CCK2R, PIK3R3	Proliferation, tumor growth, tumor size	[157, 158]
miR-194	PDK1, AKT2, XIAP, MAP4K4	Proliferation, apoptosis, migration, angiogenesis, cell cycle arrest, tumor growth and size, differentiation, metastasis	[159, 160]
miR-195	BCL2	Apoptosis	[161]
miR-200a/c	ZEB1/2	EMT	[87, 162]
miR-206	NOTCH3	Proliferation, migration, apoptosis, cell cycle arrest	[163]
miR-214	FGFR1	Proliferation, migration, invasion, tumor growth, metastasis	[164]
miR-218	BMI1	Proliferation, apoptosis, cell cycle arrest	[165]
miR-224	CDC42	Migration	[166]
miR-320a	CTNNB1, RAC1	Proliferation, migration, invasion, cell cycle arrest	[167, 168]
miR-342	DNMT1	Proliferation, invasion, cell cycle arrest, tumor growth, metastasis	[169]
miR-365	BCL2, CCND1	Apoptosis	[170]
miR-375	PIK3CA	Proliferation, cell cycle arrest, tumor growth	[171]
miR-378	VIM	Proliferation, invasion, tumor growth and size, metastasis	[172]
miR-429	ONECUT2	Migration, invasion, EMT	[173]
miR-455	RAF1	Proliferation, invasion	[174]
miR-491	BCLXL	Apoptosis	[175]
miR-638	SOX2	Invasion, migration, EMT	[176]

**Table 3:** Conventional chemotherapy treatments and targeted therapy for colorectal cancer.

Treatment	Mechanism
*Drugs used in conventional chemotherapy treatments*	

5-fluorouracil (5FU)	Inhibition of nucleotide biosynthesis. Prodrug. Entering the cell through uracil transport system. Intracellular transformation into FdUMP, FdUTP, and FUTP.

Capecitabine (Xeloda)	Metabolic precursor of 5FU. Requiring the activity of carboxylesterase, cytidine deaminase, and uridine phosphorylase.

Methotrexate (MTX)	Blocking nucleotide biosynthesis as it is a potent competitive inhibitor of dihydrofolate reductase, an enzyme that participates in the tetrahydrofolate synthesis (required for de novo biosynthesis of purine and pyrimidine bases).

Leucovorin (Wellcovorin)	Calcium folinate. Enhancing 5FU activity. It is a 5-formyl derivative of tetrahydrofolic acid and is readily converted to other reduced folic acid derivatives (e.g., tetrahydrofolate). It does not require dihydrofolate reductase activation and may activate this enzyme and is used in rescue therapies after methotrexate treatment.

Oxaliplatin (Eloxatin)	Platinum containing compound that form inter- and intrastrand crosslinks in DNA, blocking replication and transcription. Approved by the FDA in 2002.

Cisplatin (Platinol, CDDP)	Platinum containing compound that form inter- and intrastrand crosslinks in DNA, blocking replication and transcription.

Irinotecan (CPT-11) (Camptosar)	Camptothecin derived prodrug. Irinotecan is activated by hydrolysis to SN-38, an inhibitor of topoisomerase I and, thus, blocks replication and transcription.

Regorafenib (Stivarga)	Multikinase inhibitor which targets angiogenic, stromal, and oncogenic receptor tyrosine kinase (RTKs). Approved by the FDA in 2012.

*Combined chemotherapy regimens*	

FOLFOX	Leucovorin (folinic acid) + 5FU + oxaliplatin (stages III & IV). Approved by the FDA in 2002 for refractory tumors and in 2004 for first-line treatments of metastatic colorectal cancer.

CapOX (XELOX)	Capecitabine + oxaliplatin (stages III & IV).

FOLFIRI (IFL)	Leucovorin (folinic acid) + 5FU + irinotecan (stage IV).

FOLFIRINOX	Leucovorin (Folinic acid) + 5FU + irinotecan + oxaliplatin (stage IV).

FL	5FU + leucovorin (folinic acid). Approved by the FDA in 1991 for first-line treatment of metastatic colorectal cancer.

*Monoclonal antibodies for targeted therapy of metastatic CRC*	

Cetuximab (Erbitux)	Cetuximab is a recombinant, human-mouse chimeric IgG1 monoclonal antibody that binds specifically to the extracellular domain of the human epidermal growth factor receptor (EGFR) on both normal and tumor cells and competitively inhibits the binding of epidermal growth factor (EGF) and other ligands, such as transforming growth factor *α*. Approved by the FDA in 2004 for use, in combination with irinotecan, for the treatment of EGFR-expressing, metastatic colorectal carcinoma in patients who are refractory to irinotecan-based chemotherapy. In 2012, approved for use in combination with FOLFIRI for first-line treatment of patients with wild-type K-ras.

Panitumumab (Vectibix)	Human mAb against EGFR (similar to cetuximab). Approved by the FDA in 2006 for the treatment of patients with EGFR-expressing, metastatic colorectal cancer with disease progression. on or following fluoropyrimidine-, oxaliplatin-, and irinotecan-containing chemotherapy regimens.

Bevacizumab (Avastin)	Bevacizumab is a recombinant humanized monoclonal antibody that binds to human vascular endothelial growth factor (VEGF), thereby preventing the interaction of VEGF with its receptors on the surface of endothelial cells. Approved by the FDA in 2013 for use in combination with fluoropyrimidine-irinotecan- or fluoropyrimidine-oxaliplatin-based chemotherapy for the treatment of patients with metastatic colorectal cancer.

Ziv-aflibercept (Zaltrap)	Recombinant fusion protein consisting of VEGF-binding portions from the extracellular domains of human VEGFR1 and 2, that are fused to the Fc portion of the human IgG1 immunoglobulin. It binds and inhibits VEGF-A, VEGF-B, and placental growth factor. It blocks angiogenesis and decreases vascular permeability. Approved by the FDA in 2012 for use in combination with FOLFIRI for the treatment of patients with metastatic colorectal cancer that is resistant to or has progressed following treatment with an oxaliplatin-containing regimen.

Ramucirumab (Cyramza)	Human mAb against VEGFR2. Inhibits angiogenesis by blocking the interaction between VEGF and VEGFR2. Approved by the FDA in 2015 for use in combination with FOLFIRI for the treatment of patients with metastatic colorectal cancer whose disease has progressed on a first-line regimen containing bevacizumab, oxaliplatin, and a fluoropyrimidine.

**Table 4:** miRNAs involved in chemoresistance in colorectal cancer treatments.

miRNA	Treatment	Verified targets in CRC	References
*miRNAs overexpressed in chemoresistance to conventional drugs*			
Let-7g	S-1	RAS, cyclin D, c-Myc, E2F, cytochrome c	[177]
miR-10b	5-FU	BIM	[178]
miR-19b	5-FU	SFPQ, MYBL2	[179]
miR-20a	5-FU, oxaliplatin	BNIP2	[180]
miR-23a	5-FU	APAF-1	[181]
miR-31	5-FU	?	[182]
miR-140	5-FU	HDAC	[183]
miR-148a	5-FU, oxaliplatin	?	[184]
miR-181b	S-15-FU	RAS, cyclin D, c-Myc, E2F, cytochrome c	[177]
miR192/215	5-FU	TYMS, DHFR	[185, 186]
miR-203	Oxaliplatin	ATM	[187]
miR-224	5-FU	?	[188]
miR-520g	5-FU, oxaliplatin	CDKNIA (p21)	[189]
miR-625-3p	Oxaliplatin	?	[190]

*miRNAs downregulated in chemoresistance to conventional drugs*			
miR-22	5-FU	BTG1	[191]
miR-34a	5-FU	SIRT1, E2F3, KIT, LDHA	[192–194]
miR-122	5-FU	PKM2	[195]
miR-129	5-FU	BCL2, TYMS, E2F3	[196]
miR-133a	Oxaliplatin	RFFL	[197]
miR-139-5p	5-FU	NOTCH1	[198]
miR-141/200c	Oxaliplatin	ZEB1	[199]
miR-143	5-FU, oxaliplatin	BCL2, NFKB, ERK5, IGF1R	[200, 201]
miR145	5-FU	FLI-1, RAD18	[202, 203]
miR-149	5-FU	FOXM1	[204]
miR-153	Oxaliplatin	FOXO3	[205]
miR-196a	Oxaliplatin	HOXA7, HOXB8, HOXC8, HOXD8	[206]
miR-200 cluster	5-FU	EMT-related genes	[207]
miR203	5-FU	TYMS	[208]
miR-204	5-FU	HMGA2	[209]
miR-222	Oxaliplatin	ADAM17	[188]
miR-297	Oxaliplatin	ABCC2	[210]
miR-451	Irinotecan	ABCC1	[211]
miR-497	5-FU, irinotecan	IGF1R	[212]
miR-519c	5-FU, oxaliplatin	ABCG2, ELAVL1 (HuR)	[213]
miR-1915	Oxaliplatin	BCL2	[214]

*miRNAs overexpressed in chemoresistance to monoclonal antibody-based therapies*			
miR-17-3p	Cetuximab	?	[215]
miR-31	Cetuximab	?	[216]
miR-126	Bevacizumab, XELOX	?	[217]
miR-146-3p	Cetuximab	IL1A	[215]
miR-199a-5p/375	Cetuximab	PHLPP1	[218]
miR-486-5p	Cetuximab	ARHGAP5, ST5, DOCK3, TOB1, PIK3R1	[215]

*miRNAs downregulated in chemoresistance to monoclonal antibody-based therapies*			
Let-7 family	Cetuximab, panitumumab	KRAS	[215, 219]
miR-7	Cetuximab	EGFR, RAF1, ERK1/2, AKT	[136]
